# Epigenetic deregulation of lamina-associated domains in Hutchinson-Gilford progeria syndrome

**DOI:** 10.1186/s13073-020-00749-y

**Published:** 2020-05-25

**Authors:** Florian Köhler, Felix Bormann, Günter Raddatz, Julian Gutekunst, Samuel Corless, Tanja Musch, Anke S. Lonsdorf, Sylvia Erhardt, Frank Lyko, Manuel Rodríguez-Paredes

**Affiliations:** 1 Division of Epigenetics, DKFZ-ZMBH Alliance, German Cancer Research Center, Heidelberg, Germany; 2 Faculty of Biosciences, Heidelberg University, Heidelberg, Germany; 3 Center for Molecular Biology of Heidelberg University (ZMBH), Im Neuenheimer Feld 282, 69120 Heidelberg, Germany; 4 DKFZ-ZMBH-Alliance, 69120 Heidelberg, Germany; 5 CellNetworks Excellence Cluster, Heidelberg University, 69120 Heidelberg, Germany; 6 Department of Dermatology, University Hospital, Ruprecht-Karls University of Heidelberg, Heidelberg, Germany

**Keywords:** Hutchinson-Gilford progeria syndrome, Epigenetics, Aging, Lamina-associated domains (LADs), DNA methylation, Chromatin accessibility

## Abstract

**Background:**

Hutchinson-Gilford progeria syndrome (HGPS) is a progeroid disease characterized by the early onset of age-related phenotypes including arthritis, loss of body fat and hair, and atherosclerosis. Cells from affected individuals express a mutant version of the nuclear envelope protein lamin A (termed progerin) and have previously been shown to exhibit prominent histone modification changes.

**Methods:**

Here, we analyze the possibility that epigenetic deregulation of lamina-associated domains (LADs) is involved in the molecular pathology of HGPS. To do so, we studied chromatin accessibility (Assay for Transposase-accessible Chromatin (ATAC)-see/-seq), DNA methylation profiles (Infinium MethylationEPIC BeadChips), and transcriptomes (RNA-seq) of nine primary HGPS fibroblast cell lines and six additional controls, two parental and four age-matched healthy fibroblast cell lines.

**Results:**

Our ATAC-see/-seq data demonstrate that primary dermal fibroblasts from HGPS patients exhibit chromatin accessibility changes that are enriched in LADs. Infinium MethylationEPIC BeadChip profiling further reveals that DNA methylation alterations observed in HGPS fibroblasts are similarly enriched in LADs and different from those occurring during healthy aging and Werner syndrome (WS), another premature aging disease. Moreover, HGPS patients can be stratified into two different subgroups according to their DNA methylation profiles. Finally, we show that the epigenetic deregulation of LADs is associated with HGPS-specific gene expression changes.

**Conclusions:**

Taken together, our results strongly implicate epigenetic deregulation of LADs as an important and previously unrecognized feature of HGPS, which contributes to disease-specific gene expression. Therefore, they not only add a new layer to the study of epigenetic changes in the progeroid syndrome, but also advance our understanding of the disease’s pathology at the cellular level.

## Background

The nuclear lamina is a filamentous mesh network that lines the inner nuclear membrane of metazoan nuclei. Its main component are type V intermediate filaments termed lamins, four of which, lamin A, B1, B2, and C, are expressed in metazoan cells [1, 2]. Over 180 mutations causing at least 13 different diseases, collectively called laminopathies, have been described for their corresponding genes [3]. One of the most severe laminopathies is Hutchinson-Gilford progeria syndrome (HGPS), a rare progeroid disease characterized by the early onset of age-related phenotypes including osteoporosis, alopecia, loss of body fat and hair, and atherosclerosis [4, 5]. Affected individuals are usually diagnosed within the first year of life due to a failure to thrive, and suffer from rapid disease progression with death occurring in their teens as a consequence of myocardial infarction or stroke [5, 6].

The classical form of HGPS is caused by an autosomal dominant mutation in exon 11 of the *LMNA* gene, which encodes both lamins A and C [7]. The mutation activates a cryptic splice site, causing the expression of a mutant lamin A that lacks 50 amino acids near the C-terminus while leaving lamin C unaffected [7, 8]. The resulting truncated protein, referred to as progerin, undergoes aberrant posttranslational modification and retains a farnesyl residue at its C-terminal CaaX motif, thus becoming permanently associated with the nuclear lamina and causing characteristic morphological changes [6]. progerin-expressing cells have been shown to display a wide range of cellular defects such as premature cellular senescence, increased levels of reactive oxygen species, clustering of nuclear pores, delayed DNA repair, and shortened telomeres [6, 9–14]. Interestingly, low-level progerin expression has also been found in cells from normal, aged individuals, suggesting that cryptic splicing of *LMNA* mRNA is part of normal aging as well [15–17].

HGPS cells also exhibit a number of epigenetic aberrations. Most prominently, heterochromatin markers such as the histone modifications H3K27me3 and H3K9me3, as well as heterochromatin protein 1 (HP1) and the H3K27me3 methyltransferase EZH2, have been shown to be downregulated in fibroblasts from HGPS patients [15, 18, 19]. Conversely, H4K20me3, another heterochromatin mark, has been reported to be increased in HGPS cells and after ectopic progerin expression [18, 20]. Furthermore, Hi-C experiments indicate that late-passage HGPS cells lose the spatial compartmentalization of active and inactive chromatin domains that is characteristic of healthy cells [19, 21].

Less is known about the HGPS-associated role of another key epigenetic modification, DNA methylation. An earlier comparison of BJ and HGPS skin fibroblasts using bisulfite padlock probes, a method for the targeted quantification of DNA methylation at a limited number of CpGs [22], identified 586 differentially methylated autosomal genes in HGPS [23]. Another study reported profound DNA methylation changes in a set of age-related genes in HGPS patients [24]. However, the authors used samples of adult onset, i.e., non-classical progeria, for their comparisons [24], thus leaving the question of DNA methylation alterations in classical HGPS unanswered. Finally, it was recently demonstrated that HGPS fibroblasts from some patients exhibit an increased “DNA methylation age” (an estimate of the biological age computed on the basis of the methylation status of 391 genomic loci) [25], hence suggesting a considerable degree of underlying changes. A comprehensive characterization of these aberrations in classical HGPS, however, remains to be performed.

A potential candidate for epigenetic changes in HGPS is lamina-associated domains (LADs), i.e., regions of the DNA that are in close contact with the nuclear lamina. They are considered to assist in the spatial organization of the genome and to exert a role in gene repression [26–28]. Importantly, LADs largely overlap with late-replicating regions [26, 27], are mostly gene-poor [26], and are enriched for the heterochromatin marks H3K9me2 and H3K9me3 [26–29]. Despite their heterochromatic nature, LADs are not associated with high levels of cytosine methylation. In fact, they overlap to a large extent with partially methylated domains (PMDs) [30, 31], i.e., long stretches of DNA with reduced levels of DNA methylation. Interestingly, PMDs have also been shown to undergo hypomethylation as a consequence of mitotic cell division and cell aging [32]. Whether they also become differentially methylated in HGPS is still an open question.

Here, we identify epigenetic deregulation of LADs as a central feature of the epigenetic alterations in HGPS. Using Assays for Transposase-accessible Chromatin (ATAC)-see/-seq and Infinium MethylationEPIC BeadChip-mediated DNA methylation profiling, we demonstrate that dermal fibroblasts from HGPS patients exhibit both chromatin accessibility and DNA methylation changes that are enriched at LADs. Importantly, we further show that these epigenetic alterations are associated with HGPS-specific gene expression changes. Together, our results strongly implicate epigenetic deregulation of LADs as an important and previously unrecognized feature of HGPS.

## Methods

### Samples

Primary HGPS patient (HGADFN155, HGADFN271, HGADFN188, HGADFN164, HGADFN122, HGADFN178, HGADFN167, HGADFN169 and HGADFN143) and parental skin fibroblasts (HGMDFN090 and HGFDFN168) were obtained from the Progeria Research Foundation (PRF) Cell and Tissue Bank (Boston, MA, USA). Age-matched primary skin fibroblasts (GM05659, GM02036, GM01864, and GM00969) were obtained from the Coriell Cell Repository (Camden, NJ, USA). A detailed overview of the cells used for each experiment is given in Additional file 1: Table S1. Cells were grown in DMEM high-glucose medium supplemented with 10% fetal bovine serum (FBS) and 1% penicillin/streptomycin under standard 37 °C and 5% CO_2_ conditions. For all experiments, early-passage cells were used (Progeria Research Foundation: 8–9 passages, Coriell: 9–12 passages).

### ATAC-see

Hyperactive Tn5 transposase production and Tn5 transposome assembly using Atto-590N-labeled oligonucleotides were carried out as described previously [33]. Cells were grown on cover slips until 70–80% confluence and fixed with 3.8% paraformaldehyde (PFA) for 10 min at room temperature. They were then permeabilized with lysis buffer (10 mM TRIS-HCl pH 7.4, 10 mM NaCl, 3 mM MgCl_2_, 0.01% IGEPAL CA-630), washed with 1× phosphate buffered saline (PBS) twice, and incubated with the transposome mixture (100 nM assembled Tn5-Atto-590N-transposomes, 25 μl Nextera tagmentation buffer, ddH_2_O to 50 μl) for 30 min at 37 °C. Subsequently, cover slips were washed three times (15 min each) with 1× PBS containing 0.01% SDS and 50 mM EDTA at 55 °C and immunostained. Imaging was performed using an Olympus FluoView FV1000 microscope. For each sample, 50 cells from three technical replicates were analyzed to determine nuclear malformation and the presence of ATAC-see foci. A nucleus was scored as malformed when showing lobulation characteristic of HGPS cells.

### ATAC-seq

ATAC-seq was performed as described previously [34]. The generated datasets are available in the GEO repository under the accession code GSE150138, (https://www.ncbi.nlm.nih.gov/geo/query/acc.cgi?acc=GSE150138) [35]. Briefly, 50,000 cells were washed with ice-cold 1× PBS and resuspended in 50 μl lysis buffer (10 mM TRIS-HCl pH 7.4, 10 mM NaCl, 3 mM MgCl_2_, 0.1% IGEPAL CA-630). The lysis reaction was carried out while spinning down the samples at 500*g* for 10 min at 4 °C. Samples were then resuspended in transposition buffer (25 μl 2× TD buffer (Illumina), 2.5 μl TDEI (Tagment DNA Enzyme, Illumina), and 22.5 μl ddH_2_O) and incubated for 30 min at 37 °C. Subsequently, samples were purified using the MinElute PCR Purification kit (Qiagen). Final libraries were then PCR-amplified, purified once again with the same kit, and subjected to paired-end sequencing on a HiSeq 4000 platform (Illumina).

Reads were trimmed by removing stretches of bases having a quality score of < 30 at the ends of the reads. The reads were mapped using Bowtie 2 [36] against the hg19 assembly of the human genome. Peaks were called using MACS2 [37] and differential peaks were quantified by DESeq2 [38].

Distribution of significant, non-sex chromosome-associated ATAC-seq peaks across the genome was determined using the subsetByOverlaps and Genomic Regions functions with the TxDb.Hsapiens.UCSC.hg19.knownGene Bioconductor annotation package (version 3.2.2) in R (version 3.3.1). The overlap with histone modifications and lamin A was calculated in the same way using previously published ChIP-seq datasets from ENCODE (https://www.encodeproject.org/) [39, 40] (ENCSR000ARX, ENCSR000ARV, ENCSR000APR, ENCSR000APN, ENCSR000APP, ENCSR000APQ, ENCSR000APO) and GEO (https://www.ncbi.nlm.nih.gov/geo/) (GSE54334) repositories [41–43]. The overlap with lamin B was tested using data from Guelen et al. [26]. The significance of ATAC-seq peak enrichment in the respective regions was assessed using Fisher’s exact test in R. Poised enhancers for dermal fibroblasts were defined as regions containing pairs of H3K4me1 peaks in close proximity (< 1500 bp) using ENCODE [39, 40] ChIP-seq data (ENCSR000ARV); active enhancers were obtained directly from the same repository (ENCSR871EJM). Transcription factor binding sites enriched in significant, non-sex chromosome-associated ATAC-seq peaks were determined using the HOMER motif analysis tool [44] against Hg19 background and the following parameters: -size: 2000, -hist: 20.

### DNA methylation analysis

DNA methylation profiles were generated using Infinium MethylationEPIC BeadChips (Illumina), following the manufacturer’s instructions. These datasets are available in the GEO repository under the accession code GSE149960 (https://www.ncbi.nlm.nih.gov/geo/query/acc.cgi?acc=GSE149960) [45]. Methylation data analysis was carried out using the R Bioconductor package Minfi (v1.20.2) [46]. Specifically, raw .IDAT files were read and preprocessed. Methylation loci (probes) were filtered for high detection *P* value (*P* > 0.01, as provided by Minfi), location on sex chromosomes, ability to self-hybridize, and potential single nucleotide polymorphism (SNP) contamination. Array normalization was performed using the *preprocessFunnorm* function, available in Minfi [46]. Quality control was performed after every preprocessing step. Subsequently, differentially methylated probes were identified by fitting a linear model followed by statistical analysis using an empirical Bayes method to moderate standard errors. Lastly, differentially methylated probes were filtered by significance threshold (*P* < 0.05, *F*-test, after correction for multiple testing using the Benjamini-Hochberg method).

For consensus clustering, probe clusters were identified using the Minfi function boundedClusterMaker with a maximum cluster width of 1500 bp and a maximum gap of 500 bp. Using the ConsensusClusterPlus package [47], consensus clustering was performed with the 5000 most variable probe clusters, i.e., the 5000 probe clusters having the highest standard deviation, with the following parameters: maxK = 6, reps = 1000, pItem = 0.8, and pFeature = 1. Samples were then assigned to the optimal number of clusters, and *β* values were sorted by hierarchical clustering for visualization. Finally, a principal component analysis (PCA) was generated on the basis of the identified 5000 probe clusters with the R package FactoMineR [48]. Density distributions for all probe clusters, as well as those overlapping with gene bodies and intergenic regions, were created using the “density” function in R. The genes containing the 2248 gene body-associated probe clusters were then identified using the R package GenomicRanges, matched with their expression values in HGPS and control fibroblasts and plotted using the heatmap.2 function in R.

For the comparison of LAD- and “solo-WCGW” CpG probe methylation levels between HGPS and control samples, we used previously published locations of lamin A LADs [41], lamin B LADs [26], and “solo-WCGWs” CpGs [32]. DNA methylation in regions overlapping with histone modifications was determined using previously published ENCODE [39, 40] data for dermal fibroblasts (ENCSR000ARX, ENCSR000ARV, ENCSR000APR, ENCSR000APN, ENCSR000APP, ENCSR000APQ, ENCSR000APO). The significance of methylation differences between HGPS and control groups was assessed using Welch’s two-sample *t*-test in R, while the significance of probe enrichment in the respective regions was assessed using Pearson’s chi-squared test with Yates’ continuity correction in R. The enrichment of transcription factor binding sites among the differentially methylated regions was analyzed using ELMER 2.0 [49, 50] with a minimum motif quality “B” and a minimum incidence of 10. Finally, DNA methylation age estimates were obtained using a recently published algorithm (Skin&Blood Clock [25]) and corrected for passage number using a passage factor *ρ*, with *ρ* = passage number*(3.32*log(cells harvested/cells seeded)).

The enrichment of ChIP-seq and associated input for lamin A (datasets obtained from GSE57149) [41], DNMT3A and DNMT3B (datasets kindly provided by Salvador Aznar-Benitah’s laboratory) [51] in LADs of dermal fibroblasts was determined by taking aligned (Bowtie2 [36]) and processed (HOMER [44]) data from previous publications [41, 51]) and intersecting it with each lamin A LAD/inter-LAD position [41], or DESeq peaks, using bedtools “intersect.” The mean enrichment at each LAD/inter-LAD or DESeq peak for each ChIP/input was determined and the log2-fold enrichment of ChIP divided by input calculated in R. The LAD and inter-LAD (or DESeq) enrichment over input was then displayed as a boxplot using default settings in R and statistical differences were calculated using a Wilcoxon test.

DNA methylation data for dermal fibroblasts of different ages were obtained from a publically available GEO dataset (GSE52025) [52] and processed in an identical fashion to that of the HGPS fibroblasts. Specifically, after filtering for probes with a high detection *P* value (*P* > 0.01), location on sex chromosomes, ability to self-hybridize, and SNP contamination, array normalization was performed using the *preprocessFunnorm* function, available in Minfi [46]. DNA methylation levels in regions overlapping lamin A LADs or H3K9me3 were then identified using previously published locations of lamin A LADs (GSE54334) [41–43] and ENCODE [39, 40] data for H3K9me3 (ENCSR000ARX), respectively, and plotted for each sample.

Werner syndrome (WS) was analyzed using a publically available GEO dataset (GSE131752) that includes methylation data for lymphocytes from 18 WS patients and 24 controls [53]. After removal of probes with a detection *P* value of more than 0.01, methylation values were averaged for each group and ranked according to the absolute difference between both groups to determine the 1000 probes with the strongest methylation differences. WS-specific lamin A LAD probe methylation was assessed using previously published locations of lamin A LADs (GSE54334) [41–43]. The significance of methylation differences between the WS and control group was tested using Welch’s two-sample *t*-test in R.

### DNA fluorescence in situ hybridization (FISH)

Bacterial artificial chromosomes (BACs) were obtained from the Children’s Hospital Oakland Research Institute (CHORI) (Oakland, CA, USA). Additional file 1: Table S2 contains a list with all BACs used and the genes they cover. BACs were labeled with the ENZO Nick translation DNA labeling system 2.0 (ENZO) using SEEBRIGHT Red 580 dUTP (ENZO) and SEEBRIGHT Green 496 dUTP (ENZO), following the manufacturer’s instructions. Subsequently, 500 ng of each probe were precipitated with 5 μl of Cot-1 DNA (1 mg/ml) (Invitrogen) and resuspended in 15 μl of hybridization buffer (10% dextran sulfate, 50% formamide, 2× SSC pH 7). Ultimately, FISH was performed as previously described [54]. Slides were imaged using Zeiss Axioskop 2 and Olympus FluoView FV1000 microscopes. For each probe, images were acquired for 60 cells. Distance measurements were performed with Fiji [55, 56] using confocal sections with a clear FISH signal. For group-specific comparisons, the respective HGPS and control sample data were pooled and subjected to Welch’s two-sample *t*-test with a 95% confidence interval in R.

### Immunostainings

For lamin A immunostainings, cells were grown on coverslips, fixed with 3.8% PFA in 1× PBS for 10 min at room temperature, and permeabilized with 0.3% Triton X-100 in 1× PBS for 15 min at room temperature. Coverslips were subsequently washed three times with 1× PBS (10 min each) and blocked with 10% FBS in 1× PBS with 0.1% (v/v) Tween 20 (PBT) for 1 h at room temperature. They were then incubated with a 1:250 dilution of lamin A/C primary antibody (sc7292, Santa Cruz) in the same solution for 90 min, washed three times with 1× PBS (10 min each), and incubated with a 1:500 dilution of Alexa 488 secondary antibody (A11017, Thermo Fisher) in the same solution for 45 min at room temperature. Finally, coverslips were washed three times with 1× PBS (10 min each), stained with DAPI, and mounted onto slides. Slides were imaged using Zeiss Axioskop 2 and Olympus FluoView FV1000 microscopes. For quantification of malformed nuclei, severely misshapen nuclei (on the basis of significant blebbing and wrinkling) were determined in three technical replicates with 100 cells counted per replicate.

### RNA-seq

RNA was isolated using TRIzol (Invitrogen) and following the manufacturer’s instructions. Total RNA was then purified with the RNA Clean & Concentrator-5 kit (Zymo Research) and reverse-transcribed using SuperScript III reverse transcriptase (Invitrogen), again following the manufacturer’s instructions. Libraries were prepared with the TruSeq RNA sample preparation kit (Illumina) and sequenced on a HiSeq 4000 machine (Illumina) using 50-bp single reads.

Reads were trimmed by removing stretches of bases having a quality score of < 30 at the ends of the reads. Then they were mapped using Tophat 2.0.6 [57] against the hg19 assembly of the human genome. Differential expression was quantified using DESeq2 [38] and Cuffdiff 2.0 [58] and subjected to multiple testing corrections. Genes with a *q*-value smaller than 0.05 were considered differentially expressed. All files related to these experiments are available in the GEO database (GSE150138, https://www.ncbi.nlm.nih.gov/geo/query/acc.cgi?acc=GSE150138) [35].

Gene ontology (GO) analyses were performed using the AmiGO 2 database [59–61]. TRANSFAC analyses were carried out using the TRANSFAC® Public 6.0 database in Match - 1.0 [62]. Gene Set Enrichment Analysis (GSEA) [63] was performed utilizing the RNA sequencing datasets of HGPS and control fibroblasts and the “hallmark” (v5.0, Arthur Liberzon [64], Broad Institute) and “KEGG” (KEGG (Kyoto Encyclopedia of Genes and Genomes)) collections from the Molecular Signature Databases (MSigDB) supplemented with the “NRF2_01” (v6.0, Xiaohui Xie, Broad Institute) and “AP1_01” (v6.0, Xiaohui Xie, Broad Institute) gene sets, using the parameters “gene_set permutation” and “1000 permutations”. Gene signatures with a false discovery rate (FDR) *q*-value of < 0.05 were considered enriched.

The comparison of HGPS gene expression patterns with those of normal fibroblasts was carried out using a publically available GEO dataset (GSE113957) [65]. After exclusion of the HGPS samples included in this dataset, fibroblast data were classified as young (< 26, *n* = 36) or old (> 60, *n* = 48) based on donor age, and expression was averaged on a per-gene basis for each group. Group-specific expression was then plotted against HGPS-specific expression for all expressed genes, as well as for those found to be differentially (*q*-value< 0.05) expressed in HGPS. Finally, Pearson correlation coefficients were computed with standard settings in R.

### Ectopic expression of progerin in control fibroblasts

Progerin cDNA was generated by standard PCR techniques and cloned into the *Eco*RI and *Bam*HI restriction sites of the expression vector pLVX-IRES-ZsGreen1 (Clontech). For viral infection of control fibroblasts, lentiviral particles were generated cotransfecting the resulting expression vector in 293T cells (ATCC), together with the packaging vectors psPAX and pMD2.G (Sigma-Aldrich). After 48 h, filtered supernatants with the lentiviral particles were collected and added to the control fibroblasts during 36 h, when ZsGreen1-expressing cells could be observed. Culture medium was then changed and fibroblasts were grown for additional 4 days.

## Results

### ATAC-see and ATAC-seq reveal genome-wide chromatin accessibility changes enriched in lamina-associated domains (LADs)

We obtained primary skin fibroblasts from nine progeria patients and six controls (Additional file 1: Table S1). We tested all these cell lines for characteristic nuclear morphology changes and progerin expression at the transcript and protein levels. HGPS fibroblasts exhibited a wide range of nuclear malformations including characteristic wrinkling and lobulation of the nuclear lamina (Additional file 1: Fig. S1A). Importantly, the fraction of cells showing nuclear malformations ranged from 30 to 89% in HGPS cells, but only from 3 to 15% in control cells (Additional file 1: Fig. S1B). In addition, expression of progerin as a consequence of an upregulation of the ∆150 *LMNA* transcript was readily detected in all HGPS, but not in control samples (Additional file 1: Figs. S2 and S3; Additional file 2).

**Fig. 1:**
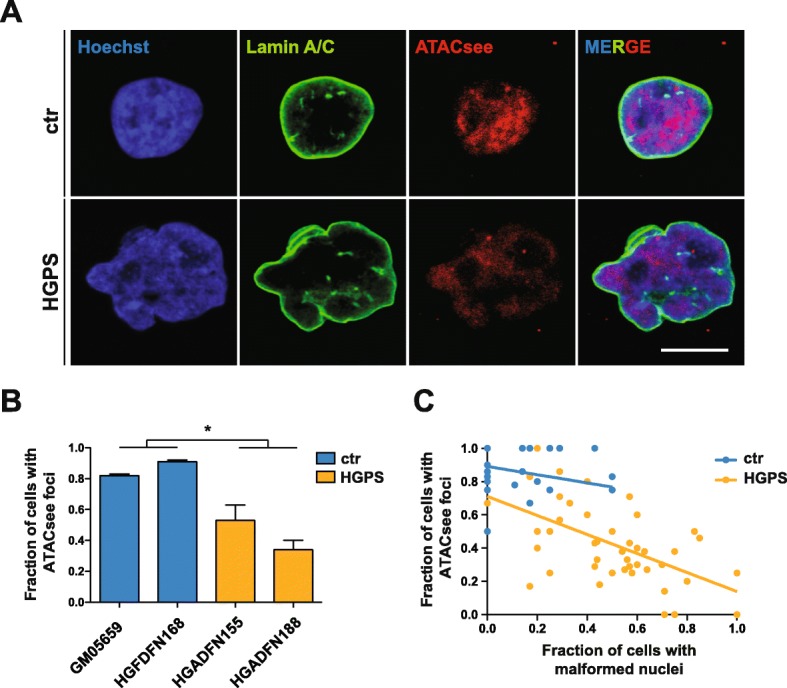
ATAC-see identifies chromatin accessibility changes in HGPS fibroblasts. **a** ATAC-see reveals loss of highly accessible chromatin foci in malformed HGPS nuclei. Scale bar = 10 μm. **b** Quantification of cells with three or more ATAC-see foci (*P* = 1.43e−2, unpaired *t*-test). Fifty nuclei were counted per sample (three replicates). **c** Correlation of the number of cells with three or more ATAC-see foci with the number of cells with malformed nuclei (HGPS: *R*^*2*^ *= 0.30*). Malformed nuclei were quantified as in **b**

In order to gain further insight into the extent of epigenetic aberrations in HGPS cells, we imaged the accessible genome in HGPS and control fibroblasts using ATAC-see [33]. Through the insertion of fluorophores by the Tn5 transposase at open chromatin sites, this technique allows the visualization of accessible chromatin using microscopy. In the large majority of control cells, we observed a high dynamic range of signal including regions of low accessibility as well as a number of bright foci representing highly accessible chromatin (Fig. 1a). Interestingly, only 34–53% of HGPS nuclei revealed these bright foci, whereas they were present in 82–91% of control nuclei (Fig. 1a, b, *P* = 1.43e−2, unpaired *t*-test). Moreover, the number of nuclei containing these foci was negatively correlated with the number of malformed nuclei in HGPS cells (*R*^2^ = 0.30), suggesting that HGPS-related nuclear malformation has an effect on chromatin accessibility (Fig. 1c).

**Fig. 2:**
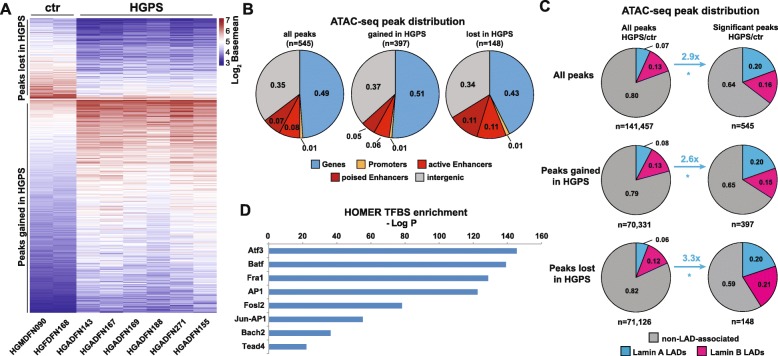
ATAC-seq reveals an enrichment of chromatin accessibility changes in lamina-associated domains (LADs). **a** Regions gaining (*n* = 397) or losing (*n* = 148) accessibility in HGPS compared with controls (*q* < 0.05, Benjamini-Hochberg). **b** Distribution of ATAC-seq peaks across genes, promoters and enhancers. **c** Distribution of ATAC-seq peaks across lamin A-, lamin B-, and non-LAD-associated regions (*P < 0.05, Fisher’s exact test). **d** HOMER transcription factor motif enrichment analysis reveals that members of the AP1 family are highly enriched (*q* < 0.01, Benjamini-Hochberg) in differentially accessible regions. Motif densities were calculated using the HOMER motif density tool for the top de novo motifs.

To further investigate this possibility and to quantify the magnitude of changes in HGPS fibroblasts, we profiled the accessible genome in HGPS cells using ATAC-seq. This identified 545 significantly differentially accessible regions between HGPS and control fibroblasts, of which 397 and 148 gained and lost accessibility in HGPS, respectively (*q* < 0.05, Benjamini-Hochberg, Fig. 2a and Additional file 1: Fig. S4). About one half of the differentially accessible regions were located in genes, about one third mapped to intergenic regions, and a minor fraction to active and poised enhancers (Fig. 2b).

Interestingly, we found several lines of evidence suggesting that genomic regions in contact with the nuclear lamina, especially those associated with lamin A, undergo alterations in chromatin accessibility in HGPS cells. First, lamin A-, but not lamin B-associated LADs were enriched 2.6- (*P* = 2.20e−16, Fisher’s exact test) and 3.3-fold (*P* < 2.20e−07, Fisher’s exact test) among the accessible regions gained and those lost in HGPS, respectively (Fig. 2c). Second, regions associated with the active chromatin marks H3K4me3 (*P* < 2.20e−16), H3K27ac (*P* = 1.27e−03), and H3K36me3 (*P* = 3.15e−06) were significantly underrepresented among the differentially accessible regions, whereas regions associated with the repressive chromatin mark H3K9me3 (*P* = 5.61e−04) were slightly overrepresented (Fisher’s exact test for all, Additional file 1: Fig. S5). Third, we observed that the relatively gene-poor chromosome 18, which tends to be located near the nuclear periphery and shows multiple LAD contacts in proliferating cells [66, 67], exhibited over seven times more differentially accessible regions than the similarly sized, gene-rich, and more centrally located chromosome 19 (Additional file 1: Fig. S6). Finally, we found that binding sites of members of the Activator Protein 1 (AP1) family of transcription factors, which have previously been shown to be associated with the nuclear lamina in mammalian cells [68], are highly enriched in the differentially accessible regions (*q* < 0.01, Benjamini-Hochberg, Fig. 2d and Additional file 1: Fig. S7). As Fos/Jun transcription factors regulate a wide range of cellular processes including cell proliferation, cellular differentiation, and apoptosis [69, 70], this is likely to have functional implications in HGPS cells. Taken together, our ATAC-see and ATAC-seq experiments provide evidence that the chromatin accessibility landscape is substantially altered in HGPS cells, with lamin A-associated LADs being at the center of these changes.

### DNA methylation profiling in HGPS reveals two patient subgroups and LAD-enriched hypermethylation

Loss of DNA methylation in lamina-associated, late-replicating regions known as partially methylated domains (PMDs) has recently been shown to constitute a pan-tissue biomarker of cellular aging [32]. To investigate whether the LAD-specific chromatin accessibility changes in HGPS cells are accompanied by alterations in DNA methylation, we performed DNA methylation profiling using Infinium MethylationEPIC BeadChips, which capture the methylation status of more than 850,000 CpGs in the human genome.

**Fig. 3:**
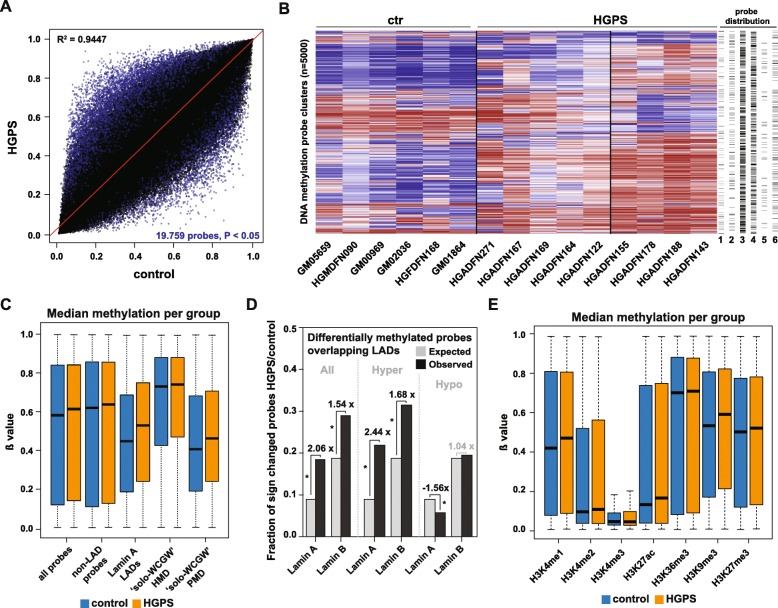
DNA methylation profiling in HGPS reveals two patient subgroups and lamina-associated domain (LAD)-enriched hypermethylation. **a** Scatter plot comparing the methylomes of HGPS and control fibroblasts. Differentially (*P* < 0.05, *F*-test) methylated probes are shown in blue. **b** Consensus clustering based on the 5000 most variable probe clusters between HGPS and control samples (1 = CpG islands, 2 = promoter, 3 = gene body, 4 = intergenic, 5 = lamin A-assoc., 6 = lamin B-assoc.). *β* values are colored from blue (*β* = 0) to red (*β* = 1). **c** Differential (*β* value) methylation of all (*P* < 2.20e−16), non-LAD- (*P* < 2.20e−16), lamin A LAD- (*P* < 2.20e−16), solo-WCGW HMD- (*P* = 4.42e−15), and solo-WCGW PMD- (*P* < 2.20e−16) associated probes (all: Welch’s two-sample *t*-test) with median indicated as a black line. HMD = highly methylated domain, PMD = partially methylated domain. **d** Enrichment of LAD-associated probes among all differentially (*P* < 0.05, *F*-test) methylated probes, as well as those hyper- and hypomethylated in HGPS samples (*P < 0.01, chi-squared test). Expected numbers were calculated based on the fraction of LAD-associated probes among all probes normalized to the number of differentially methylated probes. Sign = significantly. **e** Differential (*β* value) methylation of probes overlapping with different histone modifications. Median indicated as a black line. H3K4me1: *P* < 2.20e−16, H3K4me2: *P* < 2.20e−16, H3K4me3: *P* < 2.20e−16, H3K27ac: *P* < 2.20e−16, H3K36me3: *P* < 8.68e−15, H3K9me3: *P* < 2.20e−16, H3K27me3: *P* < 2.20e−16 (all: Welch’s two-sample *t*-test). Outliers were hidden for H3K4me3

After batch correction and removal of sex chromosome-associated probes, we identified 19,759 differentially methylated (*P* < 0.05, *F*-test) probes between nine HGPS and six control samples (Fig. 3a). Unsupervised consensus clustering of the 5000 most variably methylated probe clusters separated the samples into three groups: while the controls formed one uniform group, the HGPS samples were split into two subgroups (Fig. 3b and Additional file 1: Fig. S8). This substructure was not associated with patient age, body site of sampling, sex, strength of progerin expression, or passage number. However, it was in agreement with a subclassification of HGPS samples based on a recently reported DNA methylation age acceleration in some HGPS patients [25]. When we applied the corresponding age estimator to our sample set, the samples were divided into an age-accelerated group (median ∆ age = 9.73 years) and a group with a slight age deceleration (median ∆ age = − 1.51 years) (Additional file 1: Fig. S9), which overall matched the two subgroups identified through consensus clustering and the previous report [25]. Further analysis of the DNA methylation patterns in the two subgroups (which did not present differences in the proportion of misshapen nuclei) revealed that, in comparison with the non-accelerated group, age-accelerated samples exhibited higher levels of partial DNA methylation across all 5000 probes, as well as gene body-associated and intergenic probes (Additional file 1: Fig. S10A). Interestingly, gene body-associated probes were distributed across 1336 genes that were enriched for developmental processes (Additional file 1: Figs. S10B and C).

Most of the 5000 most variable probe clusters used for the subgrouping contained probes located in genes and intergenic regions, while only a few were located in CpG islands and promoters (Fig. 3b, right panel). Together with the fact that CpG island methylation in the HGPS samples was virtually unchanged (Additional file 1: Fig. S11A), this indicates that aberrant CpG island methylation, a prominent epigenetic alteration of normal cells during aging [71, 72], plays only a minor role in HGPS. We also tested for the enrichment of transcription factor binding sites among the regions comprising the differentially methylated probes in HGPS using ELMER. In agreement with our ATAC-seq findings, AP1 family members were the most enriched in these regions (odds ratio > 2.1, 95% confidence interval, Additional file 1: Fig. S12), confirming that this transcription factor family is strongly associated with the epigenetic changes in HGPS fibroblasts.

One of the major epigenetic alterations known in HGPS is the loss of heterochromatin-associated histone marks at the nuclear periphery of progerin-expressing cells [15, 18, 19]. We therefore tested whether DNA methylation is similarly altered in genomic regions that are in contact with the nuclear lamina. While we only found a minor increase in median methylation levels of all 850,000 EPIC probes as well as non-LAD probes, lamin A LAD-associated probes exhibited a strong and significant (*P* < 2.20e−16, Welch’s two-sample *t*-test) increase (Fig. 3c) and were enriched 2.06-fold among the differentially methylated probes in HGPS (*P* < 2.20e−16, chi-squared test, Fig. 3d). Interestingly, this enrichment was caused by a significant overrepresentation of lamin A LAD-associated hypermethylated probes (2.44-fold, *P* < 2.20e−16, chi-squared test) among differentially methylated probes, whereas hypomethylated probes were slightly, but significantly underrepresented (− 1.56-fold, *P* < 2.35e−13, chi-squared test) (Fig. 3d). Lamin B LAD-associated probes, while overrepresented as well, were less enriched (1.54-fold, *P* < 2.20e−16, chi-squared test) and showed less pronounced methylation changes (Fig. 3d and Additional file 1: Fig. S11B).

A recent study identified a subset of lamina-associated CpGs in PMDs (termed “solo-WCGWs”), which track the mitotic history of a cell and gain substantial age-related hypomethylation in a wide range of tissues [32]. Given the occurrence of different aging phenotypes in HGPS cells, we wondered whether these “solo-WCGWs” CpGs show similar hypomethylation in HGPS. Intriguingly, median methylation levels of PMD-associated “solo-WCGWs” were not reduced in HGPS, but significantly increased compared to control cells (*P* < 2.20e−16, Welch’s two-sample *t*-test, Fig. 3c). In comparison, methylation levels of “solo-WCGW” probes associated with highly methylated domains (HMDs) exhibited only minor changes (Fig. 3c). We conclude that, despite the progeroid phenotype of HGPS fibroblasts, age-related hypomethylation of PMDs is not a feature of the HGPS-specific DNA methylome.

To determine more directly whether HGPS-specific DNA methylation changes differ from those observed during normal aging, we obtained data from a previous study of DNA methylation in untransformed human fibroblasts originating from donors aged 23–63 years [52]. Despite considerable variation between samples, median DNA methylation levels in regions overlapping with H3K9me3 or lamin A LADs tended to decline with increasing donor age (Additional file 1: Figs. S13A and B), thus reinforcing the notion that HGPS-specific DNA methylation pattern are distinct from those occurring during physiological aging.

Further leveraging publicly available ChIP-Seq datasets from dermal fibroblasts, we expanded our analysis of DNA methylation changes in genomic regions overlapping various histone modifications. Genomic regions marked by H3K4me1 and H3K9me3 showed the strongest HGPS-specific increases in median DNA methylation levels (*P* < 2.20e−16, Welch’s two-sample *t*-test, Fig. 3e), resembling those seen in lamin A LAD-associated probes (Fig. 3c). Consistently, differentially (*P* < 0.05, *F*-test) methylated probes were enriched in regions overlapping H3K4me1 (1.38-fold) and H3K9me3 (1.60-fold) and underrepresented in regions associated with H3K4me3 (− 2.54-fold) and H3K36me3 (− 1.33-fold) (chi-squared test for all, Additional file 1: Fig. S14A). Interestingly, H3K9me3-associated probes were only enriched among hypermethylated (1.76-fold, chi-squared test), but not hypomethylated probes, indicating that regions marked by H3K9me3 in dermal fibroblasts predominantly gain DNA methylation in HGPS (Additional file 1: Figs. S14B and C). Using a previously published ChIP-seq dataset [51], we also noticed that DNMT3B, but not DNMT3A, is significantly enriched in genomic regions associated with lamin A (*P* < 2.20e−16, Wilcoxon test, Additional file 1: Fig. S15), thus providing a potential mechanistic link to the observed DNA methylation changes in HGPS cells.

Finally, another progeroid disease, Werner syndrome (WS), has recently been shown to result in changes in DNA methylation patterns and accelerated DNA methylation age [53, 73]. Using an available dataset [53], we therefore tested whether WS methylomes are characterized by alterations similar to those of HGPS methylomes. In contrast to our findings for HGPS cells, DNA methylation levels were highly correlated between 18 WS and 24 control samples (Additional file 1: Figs. S16A and B) and showed no lamin A LAD-associated DNA hypermethylation (Additional file 1: Fig. S16C). Although it is necessary to point out that the analyzed WS methylomes were generated from lymphocytes and not dermal fibroblasts, these results indicate that the epigenetic deregulation of LADs is a feature unique to the HGPS methylome.

Altogether, these results suggest that DNA methylation alterations in HGPS, while distinct from those observed during healthy aging or WS, are not randomly distributed, but occur primarily in regions that are lamina-associated, partially methylated and characterized by the presence of heterochromatic histone marks in normal dermal fibroblasts.

### Epigenetic deregulation of LADs contributes to aberrant gene expression in HGPS

**Fig. 4:**
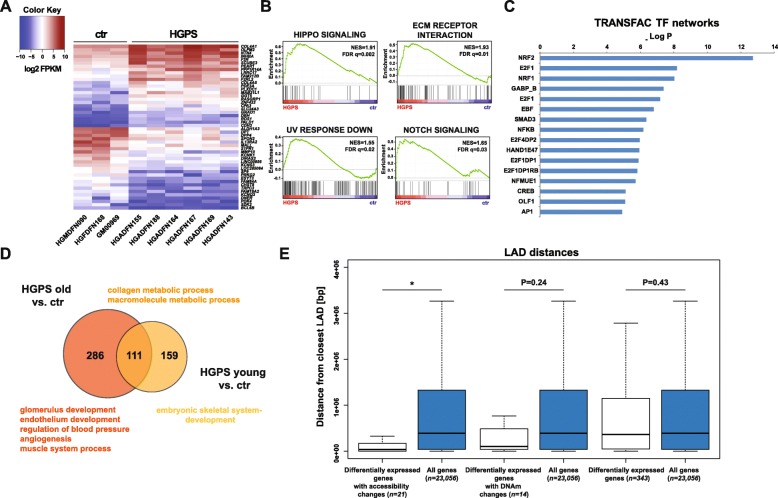
Epigenetic deregulation of lamina-associated domains (LADs) contributes to aberrant gene expression in HGPS. **a** 50 most differentially (*q* < 0.05, Benjamini-Hochberg) expressed genes in six HGPS vs. three control samples. Lowly expressed genes are shown in blue, highly expressed ones in red. FPKM = fragments per kilobase of transcript per million mapped reads. **b** Selection of Gene Ontology (GO), Kyoto Encyclopedia of Genes and Genomes (KEGG) and hallmark gene sets enriched (false discovery rate (FDR) *q* < 0.05) in HGPS fibroblasts. NES = normalized enrichment score. **c** TRANSFAC transcription factor (TF) network analysis of upstream factors controlling the observed expression changes. **d** Venn diagram showing numbers of genes overlapping between HGPS young (< 8 years) vs. control samples and HGPS old (> 8 years) vs. control samples, respectively. The GO processes characteristic of each comparison are given. **e** Median distance to the nearest LAD (lamin A and/or lamin B) for the indicated sets of genes. Numbers of genes in each group and Wilcoxon rank-sum test *P* values (with continuity correction) are given. *P = 2.25e−4. DNAm = DNA methylation

To investigate whether the identified LAD-specific chromatin accessibility and DNA methylation changes contribute to aberrant gene expression in HGPS cells, we performed RNA sequencing (RNA-seq) using fibroblasts from six HGPS patients and three controls. A total of 343 genes were found to be significantly (*q* < 0.05, Benjamini-Hochberg) differentially expressed between HGPS and control cells, of which 160 were upregulated and 183 were downregulated in HGPS, respectively (Fig. 4a). Importantly, many of these genes, especially those more strongly deregulated, overlapped with genes previously identified to be differentially expressed in HGPS [11, 74] (Additional file 1: Fig. S17).

Gene Ontology (GO) analysis suggested “organismal” and “developmental” processes as well as “signaling” and “cell communication” as the main pathways to be affected by these changes (Additional file 1: Fig. S18). In addition, Gene Set Enrichment Analyses (GSEA) highlighted Hippo signaling, ECM receptor interaction, UV response and Notch signaling, among others, to be significantly overrepresented in the set of differentially regulated genes (Fig. 4b and Additional file 1: Fig. S19). GSEA analyses also revealed a slight enrichment of AP1 target genes, even though it did not reach statistical significance (normalized enrichment score (NES) = 1.05, FDR *q*-val = 0.285, Additional file 1: Fig. S19). TRANSFAC analysis of putative upstream regulators identified NRF2 as the main transcription factor associated with these changes (Fig. 4c). Importantly, NRF2 has previously been reported to be sequestered to the nuclear lamina by progerin and to cause a subset of HGPS-associated expression changes [11].

Given the rapid progression of age-related pathologies in HGPS patients, we also wondered whether cells from young and older patients differ significantly with regard to their expression patterns. Indeed, deregulated genes in younger patients (< 8 years) were associated with the GO terms “embryonic skeletal system development” and “anterior/posterior pattern specification” (Fig. 4d and Additional file 1: Fig. S20A). In comparison, differentially expressed genes in older patients (> 8 years) were associated with typical HGPS-related pathological features including “glomerulus development,” “endothelium development,” “regulation of blood pressure,” “angiogenesis,” and “muscle system process” (Fig. 4d and Additional file 1: Fig. S20B). In conclusion, both HGPS-related developmental changes and the aggravation of the clinical phenotype can be recapitulated at the level of gene expression in vitro.

We also compared HGPS-specific expression changes to those of aging normal dermal fibroblasts analyzed in a previous study [65]. Interestingly, while overall HGPS fibroblast-specific gene expression correlated well with those of both young (< 26 years) and old fibroblasts (> 60 years) (both: *R*^2^ = 0.81, Additional file 1: Figs. S21A and B), expression of the 343 HGPS-specific differentially expressed genes in old fibroblasts was more similar to that of control fibroblasts (*R*^2^ = 0.83) than to HGPS fibroblasts (*R*^2^ = 0.58, Additional file 1: Figs. S21B and C). These findings indicate that HGPS-specific gene expression patterns are distinct from those observed during physiological aging.

In order to assess whether LAD-associated epigenetic changes contribute to the gene expression patterns observed in HGPS fibroblasts, we compared the RNA-seq datasets with the DNA methylation and ATAC-seq datasets. Of the 343 genes with significant expression changes, 21 showed simultaneous changes in accessibility in HGPS (Additional file 1: Fig. S22A). In comparison, simultaneous DNA methylation changes were observed in 14 genes (Additional file 1: Fig. S22B), and three of the differentially expressed genes (*EDIL3*, *RELN*, and *ZNF423*) were both differentially methylated and differentially accessible. Interestingly, the 21 genes undergoing both accessibility and expression changes are localized in or in close proximity to LADs in the genome of dermal fibroblasts (*P* = 2.25e−4, Wilcoxon rank-sum test, Fig. 4e). Although not reaching statistical significance, a similar trend was observed for the 14 genes undergoing both DNA methylation and expression changes (*P* = 2.43e−1, Wilcoxon rank-sum test, Fig. 4e). These findings reveal an important subset of differentially expressed genes, which is affected by the epigenetic deregulation of LADs in HGPS.

**Fig. 5:**
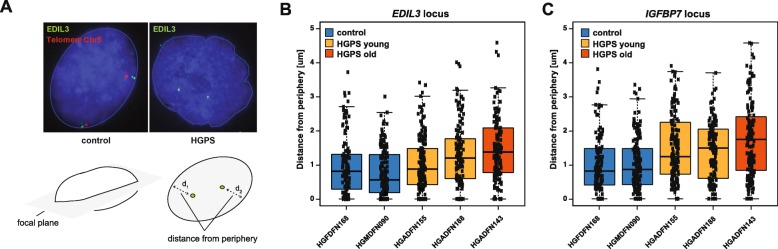
Epigenetic deregulation is accompanied by intranuclear relocalization in HGPS. **a** Representative *EDIL3* FISH images in HGPS and control nuclei. A telomeric probe (red) on Chr5 was used as a positive staining control. The distance from the FISH signal to the nuclear periphery was measured in the focal plane in cells exhibiting a clear biallelic signal. **b**, **c** Quantification of **a** for *EDIL3* and *IGFBP7* loci in two control and three HGPS cell lines for 60 cells per sample. *EDIL3*: *P* = 3.03e−08, Welch’s two-sample *t*-test. *IGFBP7: P* = 2.75e−13, Welch’s two-sample *t*-test

To test whether the epigenetic deregulation of this subset of genes is associated with an intranuclear relocalization of the underlying genomic loci, we performed fluorescence in situ hybridization (FISH) experiments in HGPS fibroblast nuclei. More specifically, we measured the distance of specific FISH signals for a selected set of genes to the nuclear lamina in HGPS and control cells (Fig. 5a, lower panel). Two of the tested genes, *EDIL3* (*P* = 3.03e−08, Welch’s two-sample *t*-test), which encodes an integrin ligand with an important role in angiogenesis, vessel wall remodeling, and development [75, 76], and *IGFBP7* (*P* = 2.75e−13, Welch’s two-sample *t*-test), which encodes a member of the insulin-like growth factor-binding protein (IGFBP) family and is related to cellular senescence and modulation of angiogenesis [77–79], were consistently localized farther away from the nuclear periphery in HGPS compared with control cells (Figs. 5b, c), with the strongest locational changes occurring in cells from older patients. Importantly, we subsequently verified the increased expression of both genes in HGPS cells using quantitative RT-PCR (Additional file 1: Figs. S23A and B)*.* In contrast, two of the genes characterized by reduced chromatin accessibility and gene expression in HGPS, *FAM19A2* and *ADCY7* (*P* = 1.13e−03 and *P* = 1.62e−03, respectively, Welch’s two-sample *t*-test), exhibited a tendency to be relocated to the nuclear periphery in HGPS cells, despite high cell-to-cell variation (Additional file 1: Figs. S24A and B). Taken together, these findings further support the notion that intranuclear relocalization of lamina-associated loci underpins the aberrant gene expression patterns in HGPS.

### Ectopic expression of progerin causes epigenetic deregulation of LADs in control fibroblasts

In order to find out whether progerin is sufficient to cause the epigenetic deregulation of LADs and HGPS-specific expression changes, we ectopically expressed the mutant protein in control fibroblasts by lentiviral transduction of progerin (pLVX-Prog) or empty-vector (pLVX-EV) constructs. Following successful detection of the mutant protein 4 days after transduction, as well as an incipient increase in the number of malformed nuclei (Additional file 1: Figs. S25A and B; Additional file 2), we tested the cells for DNA methylation and gene expression changes similar to those observed in HGPS fibroblasts. Importantly, cells showed a significant (*P* < 2.20e−16, Welch’s two-sample *t*-test) hypermethylation of lamin A- and lamin B-associated LADs similar to that observed in HGPS cells (Additional file 1: Fig. S25C). Consistently, a number of genes identified to be differentially expressed in HGPS fibroblasts, including *EDIL3* and *IGFBP7*, became up- or downregulated as observed in the primary cells (Additional file 1: Fig. S26). These results provide evidence that the observed epigenetic deregulation of LADs and its downstream effects are a direct consequence of progerin expression.

## Discussion

Previous studies have reported widespread histone modification changes in HGPS cells [15, 18, 19], but little is known about their effect on chromatin accessibility and their relationship to DNA methylation patterns. To explore the nature and extent of potential alterations in these regulatory layers, as well as their influence on disease-specific gene expression, we subjected primary dermal fibroblasts from different HGPS patients to an integrated analysis using ATAC-see/-seq, DNA methylation profiling, and RNA-seq. Our data reveal epigenetic deregulation of LADs as a novel, defining feature of the HGPS epigenome, which contributes to aberrant gene expression in the disease.

We found HGPS-specific chromatin accessibility and DNA methylation changes to be significantly enriched in genomic regions that are in contact with the nuclear lamina in normal dermal fibroblasts [26, 41]. This enrichment was observed for both regions gaining and losing accessibility in the disease, thus indicating considerable reconfiguration of peripheral chromatin in HGPS nuclei. Importantly, as more than two thirds of the differentially accessible regions exhibited increased accessibility in the disease, lamina-associated chromatin appears to undergo substantial relaxation, which is in agreement with the observation that progerin-expressing cells are characterized by reduced levels of the heterochromatin marks H3K9me3 and H3K27me3 in the vicinity of the nuclear lamina [18, 19]. Similarly, Hi-C experiments with HGPS fibroblasts have demonstrated a considerable loss of chromatin compartmentalization, i.e., a reduction of chromatin compartment identity, at later passages [19, 21]. Our ATAC-see-related findings are in accordance with these observations, even though the HGPS-specific decrease in ATAC-see foci seems to be in disagreement with previous reports demonstrating a loss of H3K9me3 and H3K27me3 in HGPS cells [15, 18, 19]. In fact, we noticed that the dark, inaccessible regions also become brighter, i.e., more accessible in many HGPS nuclei, thus indicating a similar loss of heterochromatic regions in our HGPS fibroblasts. However, in comparison with the decrease in ATAC-see foci, this phenomenon was less prevalent and less homogenously distributed across different cells.

The rather limited number of regions exhibiting significant chromatin accessibility changes may appear surprising, given the numerous epigenetic changes observed in HGPS cells. However, we note that lamin A/C immunostainings of HGPS fibroblasts showed high levels of population heterogeneity, with individual nuclei exhibiting a wide range of nuclear malformations. The set of regions we identified as significantly differentially accessible may therefore represent a consensus of chromatin regions shared by a minimum number of progerin-expressing cells in the population. Future experiments with single-cell resolution in primary tissues should provide a better characterization of these changes in individual cells.

LADs largely overlap with PMDs, which have been shown to exhibit reduced levels of DNA methylation as a consequence of mitotic aging [32]. Surprisingly, in HGPS cells, we observed an increase of median methylation levels in regions associated with lamin A, H3K9me3 or solo-WCGWs. While this appears contradictory to the increase in peripheral chromatin accessibility and the reported loss of the heterochromatin marks HP1, H3K9me3, and H3K27me3 in HGPS cells [15, 18, 19], it may represent a consequence of de novo DNMT activity, as DNMT3B exhibited significant enrichment in regions associated with lamin A. It is therefore tempting to speculate that the remodeling of peripheral heterochromatin in HGPS cells [21] renders the underlying regions more susceptible to DNMT3B-mediated DNA hypermethylation. Additionally, HGPS-specific relocalization of formerly LAD-associated regions within the nucleus, leading to increased expression as observed in the case of the *EDIL3* and *IGFBP7* loci, may contribute to the observed DNA methylation changes. Such a conclusion is supported by the finding that a substantial portion of the HGPS-specific DNA methylation changes occurred in gene bodies and that active gene expression positively correlates with gene body methylation [80].

Our results also indicate that considerable DNA methylation differences exist not only between HGPS and control samples, but also within the group of patient fibroblasts. Intriguingly, these are manifested in a DNA methylation age acceleration for some patients, but not others. Despite small deviations, which might be attributable to differences in the number of population doublings or data preprocessing [81], our DNA methylation age estimates closely resemble previously published data for the same samples [25]. Intriguingly, this HGPS-specific age acceleration seems to be reflected at the transcriptional level as well, as HGPS fibroblasts have recently been shown to exhibit a transcriptomic age acceleration of 9–10 years [65], which closely matches the median age difference (9.73 years) identified for one of the HGPS subgroups in our analysis (Additional file 1: Fig. S9). Of note, the methylomes of our age-accelerated fibroblast subgroup present higher partial methylation, a well-established hallmark of aging, even in gene bodies. However, we detected no or very limited differences between the two subgroups with regard to chromatin accessibility and gene expression, respectively, and the extent of these changes did not match those observed between HGPS and control cells (data not shown). While this is likely to be a consequence of the relatively low number of samples from both groups used for the comparisons, further studies are necessary to better decipher the molecular implications of the detected DNA methylation differences between individual HGPS patients.

The relatively limited effect of LAD-related chromatin accessibility and DNA methylation changes on gene expression is consistent with the observation that LADs are generally gene-poor [26] and that local alterations in the LAD landscape can cause global transcriptional changes not restricted to genes in LADs [82]. However, it also suggests that other mechanisms contribute to the aberrant gene expression of HGPS cells. In this regard, NRF2 mislocalization to the nuclear lamina has recently been demonstrated to be a key contributor to the HGPS-specific transcriptome [11]. Our data are in agreement with this finding, since we identified the transcription factor as one of the key factors responsible for the HGPS-related gene expression changes, even though its target genes did not reach statistical significance in our GSEA analyses (Additional file 1: Fig. S16). Furthermore, NRF2 binding motifs were significantly enriched in the differentially accessible regions between HGPS and control cells. Because the transcription factor has been shown to be sequestered to the nuclear lamina by progerin [11], it is tempting to speculate that the epigenetic changes observed in LADs are involved in the NRF2-mediated differential expression in the disease.

The nuclear lamina is known to be a resting place for transcription factors, serving to sequester them away from chromatin [83]. For example, c-Fos, a member of the AP1 family of transcription factors has been demonstrated to be negatively regulated by binding to lamin A/C at the nuclear lamina [68]. This function might be impaired by the presence of progerin in HGPS cells, since we found AP1 members to constitute the transcription factor family that is most affected by the epigenetic deregulation in HGPS. Intriguingly, both *IGFBP7* and *EDIL3* contain consensus binding sites for AP1 family members in their respective promoter [78] or enhancer [84], further indicating that their differential expression is likely driven by epigenetic changes in HGPS cells.

**Fig. 6:**
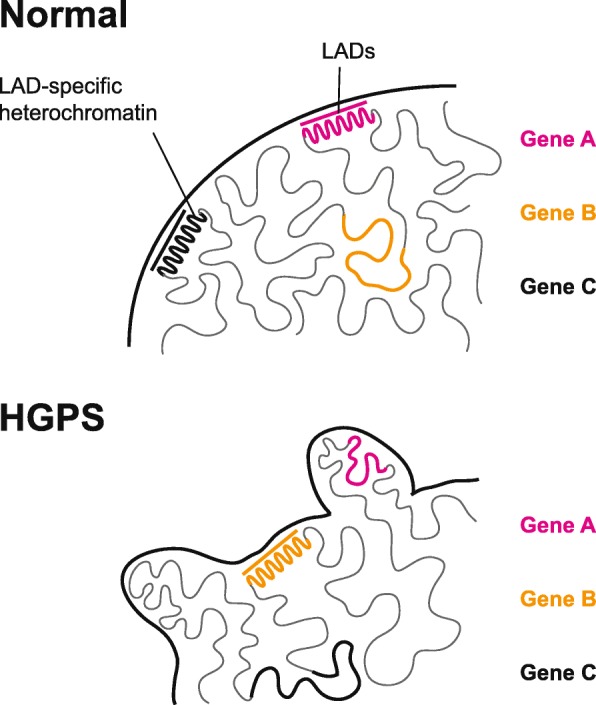
Epigenetic deregulation of lamina-associated domains (LADs) in HGPS. Progerin-driven nuclear malformation in HGPS nuclei causes substantial, but potentially locally stochastic epigenetic reconfiguration of LAD-specific chromatin. As peripheral heterochromatin is diminished in these cells, many of the affected regions gain a more relaxed chromatin environment that is more permissive to the binding of transcription factors and might thus facilitate differential expression. In some cases, chromatin decondensation and disease-specific differential expression of formerly LAD-associated loci coincides with their relocalization within the nucleus, as detected in the case of *EDIL3* and *IGFBP7*

In summary, our results suggest a scenario, in which the progerin-driven nuclear malformation of HGPS nuclei causes substantial, but potentially locally stochastic epigenetic reconfiguration of LAD-specific chromatin (Fig. 6). As peripheral heterochromatin is diminished in these cells [15, 18, 19], many of the affected regions gain a more relaxed chromatin environment that is more permissive to the binding of transcription factors and might thus facilitate differential expression. In some cases, chromatin decondensation and disease-specific differential expression of formerly LAD-associated loci accompanies their relocalization within the nucleus (Fig. 6), as detected for *EDIL3* and *IGFBP7*. Further experiments are needed to clarify whether similar mechanisms contribute to disease-specific gene expression in other tissues and in other laminopathies.

## Conclusions

We have performed an integrated analysis of chromatin accessibility, DNA methylation, and RNA expression changes in dermal fibroblasts from HGPS patients. Importantly, we identify epigenetic deregulation of LADs as a central characteristic of the HGPS-specific epigenome, as both HGPS-specific DNA methylation and chromatin accessibility changes are enriched in regions associated with the nuclear lamina. Furthermore, we demonstrate that the abovementioned alterations contribute to the aberrant gene expression pattern observed in the disease. Taken together, our data not only add a new layer to the study of epigenetic changes in the progeroid syndrome, but also advance our understanding of the disease’s pathology at the cellular level.

## Supplementary information

**Additional file 1.** Supplementary tables and figures for the manuscript.

**Additional file 2.** Original uncropped Western blots.

## Data Availability

The ATAC-seq and RNA-seq datasets generated during this study are available from the GEO repository under the accession code GSE15038 (https://www.ncbi.nlm.nih.gov/geo/query/acc.cgi?acc=GSE150138) [35]. Generated Infinium MethylationEPIC BeadChip datasets are also available from the GEO database (GSE149960, https://www.ncbi.nlm.nih.gov/geo/query/acc.cgi?acc=GSE149960) [45]. As for the publicly available ChIP-seq data used for our analyses, lamin A profiles from dermal fibroblasts were obtained from GEO datasets GSE57149 and GSE54334 [41–43], while lamin B profiles were obtained from Guelen et al [26], and DNMT3A and DNMT3B ChIP-seq datasets were kindly provided by Salvador Aznar-Benitah’s laboratory [51]. DNA methylation profiles from healthy fibroblasts of different ages and Werner syndrome (and control) lymphocytes were obtained from GEO datasets GSE52025 [52] and GSE131752 [53], respectively. Finally, gene expression data from healthy fibroblasts of different ages were obtained from the GEO dataset GSE113957 [65].

## Abbreviations

ATAC: Assay for Transposase-accessible Chromatin
FDR: False discovery rate
FISH: Fluorescence in situ hybridization
GO: Gene Ontology
GSEA: Gene Set Enrichment Analysis
HGPS: Hutchinson-Gilford progeria syndrome
HMD: Highly methylated domains
LAD: Lamina-associated domains
NES: Normalized enrichment score
PMD: Partially methylated domains
WS: Werner syndrome
