# Supplementary material for: Epigenetic deregulation of lamina-associated domains in Hutchinson-Gilford progeria syndrome
Source: Genome Med. 2020 May 25;12:46. doi: 10.1186/s13073-020-00749-y (PMC7249329; doi:10.1186/s13073-020-00749-y)
Supplement: Supplementary file 1 — Additional file 1. Supplementary tables and figures for the manuscript. [file 13073_2020_749_MOESM1_ESM.pdf]

Supplementary Material

Epigenetic deregulation of lamina-associated domains in Hutchinson-Gilford Progeria Syndrome

Florian Köhler[1,2], Felix Bormann[1], Günter Raddatz[1], Julian Gutekunst[1], Samuel Corless[3,4,5], Tanja Musch[1], Anke S. Lonsdorf[6], Sylvia Erhardt[3,4,5], Frank Lyko[1] & Manuel Rodríguez-Paredes[1]*

* Correspondence: m.rodriguez@dkfz.de

[1]Division of Epigenetics, DKFZ-ZMBH Alliance, German Cancer Research Center, Heidelberg, Germany; [2]Faculty of Biosciences, Heidelberg University, Heidelberg, Germany; [3]Center for Molecular Biology of Heidelberg University (ZMBH), Im Neuenheimer Feld 282, 69120 Heidelberg, Germany; [4]DKFZ-ZMBH-Alliance, 69120 Heidelberg, Germany; [5]CellNetworks Excellence Cluster, Heidelberg University, 69120 Heidelberg, Germany [6]Department of Dermatology, University Hospital, Ruprecht-Karls University of Heidelberg, Heidelberg, Germany

Table S1. Primary cells used in this study

PRF = Progeria Research Foundation, CCR = Coriell Cell Repository, 1 = DNA methylation profiling, 2 = ATAC-see, 3 = ATAC-seq, 4 = RNAseq, 5 = ectopic expression of Progerin

Sample ID	Status	Gender	Age [years]	Source	Experiments
HGADFN155	HGPS	Female	1.17	PRF	1,2,3,4
HGADFN271	HGPS	Male	1.25	PRF	1,3
HGADFN188	HGPS	Female	2.25	PRF	1,2,3,4
HGADFN164	HGPS	Female	4.67	PRF	1,4
HGADFN122	HGPS	Female	5.00	PRF	1
HGADFN178	HGPS	Female	6.92	PRF	1
HGADFN167	HGPS	Male	8.42	PRF	1,3,4
HGADFN169	HGPS	Male	8.50	PRF	1,3,4
HGADFN143	HGPS	Male	8.83	PRF	1,3,4
GM05659	control	Male	1.00	CCR	1,2,5
GM00969	control	Female	2.00	CCR	1,4,5
GM01864	control	Male	11.00	CCR	1
GM02036	control	Female	11.00	CCR	1
HGMDFN090	control	Female	37.83	PRF	1,3,4
HGFDFN168	control	Male	40.42	PRF	1,2,3,4

Table S2. BACs used in this study

BAC Name	Reference	Chromosome	Location
EDIL3	RP11-845G7	5	*EDIL3* gene
Chr5 control	RP11-82M24	5	telomere
IGFBP7	RP11-589G9	4	*IGFBP7* gene
RELN	RP11-57M15	7	*RELN* gene
SOX11	RP11-103F8	2	*SOX11* gene
SMAD9	RP11-354L15	13	*SMAD9* gene
KCNK1	RP11-349N15	1	*KCNK1* gene
ACTB	RP11-754B14	7	*ACTB* gene
ADCY7	RP11-321D3	16	*ADCY7* gene
FAM19A2	RP11-80D18	12	*FAM19A2* gene

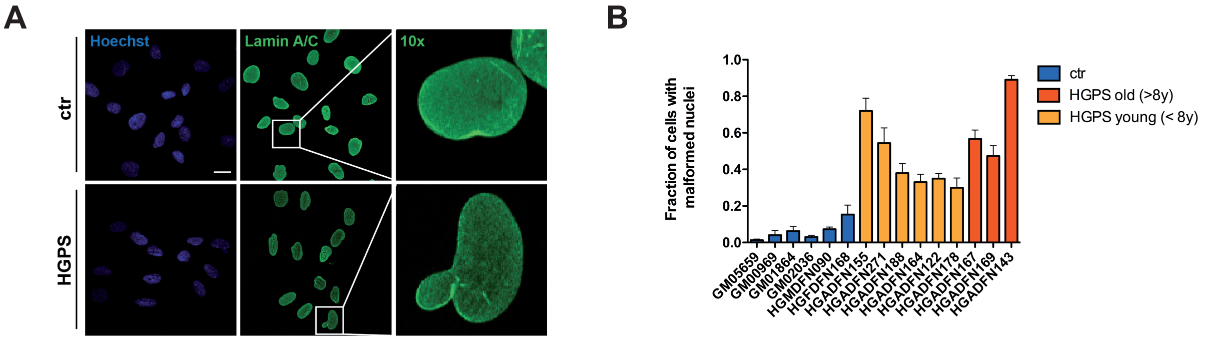

Fig. S1. Nuclear morphology in the fibroblast model system. (A) α-Lamin A/C immunofluorescence showing characteristic nuclear malformation in HGPS (HGPS: HGADFN188, ctr: HGMDFN090, scale bar = 10 µm). **(B)** Quantification of malformed nuclei in HGPS and control fibroblast cell lines. HGPS cells were grouped as young (<8 years) or old (>8 years) depending on the age of the donor (source: Progeria Research Foundation).

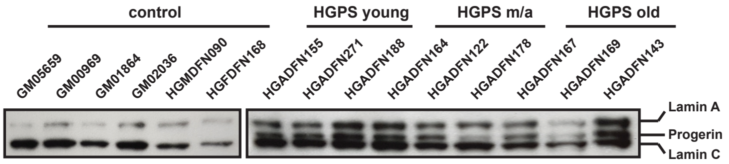

Fig. S2. Progerin protein expression in the fibroblast model system. Western Blot with α-Lamin A/C antibody (sc7292, Santa Cruz) showing expression of Progerin in HGPS samples. The band corresponding to Lamin C was used as a loading control.

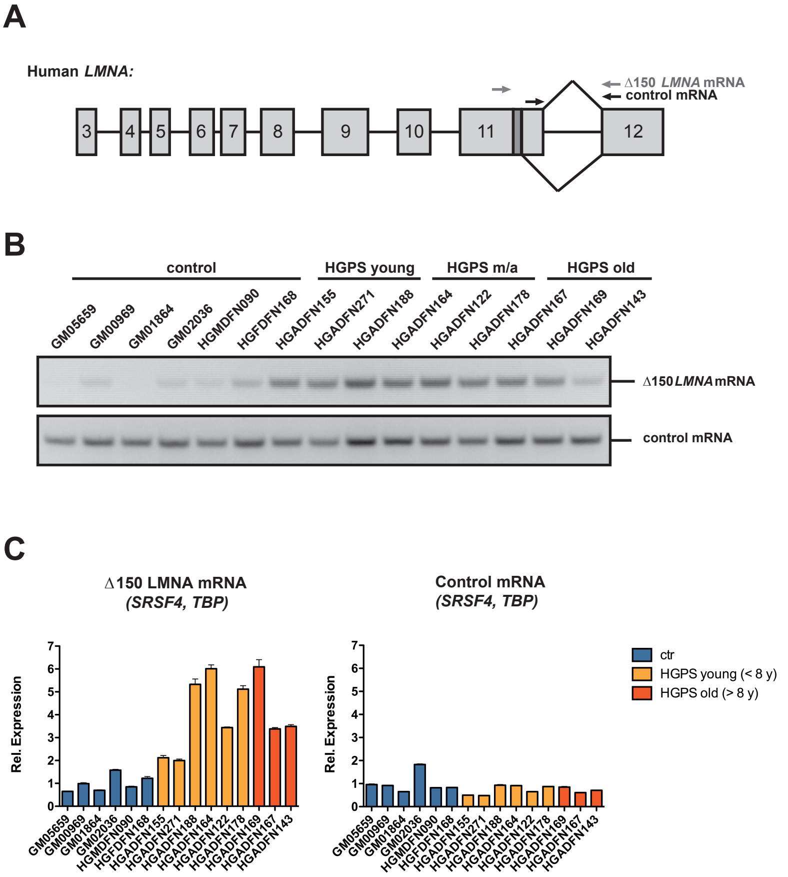

Fig. S3. Progerin transcript expression in the fibroblast model system. (A) Schematic representation of *LMNA* gene. The locations of the primers designed for the detection of the Δ150 *LMNA* and control mRNAs are indicated. **(B)** Detection of Δ150 *LMNA* mRNA in HGPS fibroblasts through RT-PCR (50 cycles). **(C)** Expression of Δ150 *LMNA* mRNA relative to *SRSF4* and *TBP* in HGPS and control fibroblasts as measured through RT-qPCR.

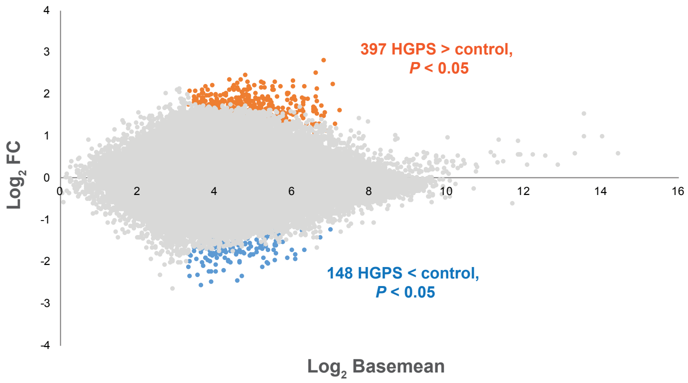

Fig. S4. Differentially accessible regions in HGPS and control. Log2 Fold Change (FC) and log2 basemean of regions gaining (n=397) and losing (n=148) accessibility in HGPS, respectively (q<0.05, Benjamini-Hochberg).

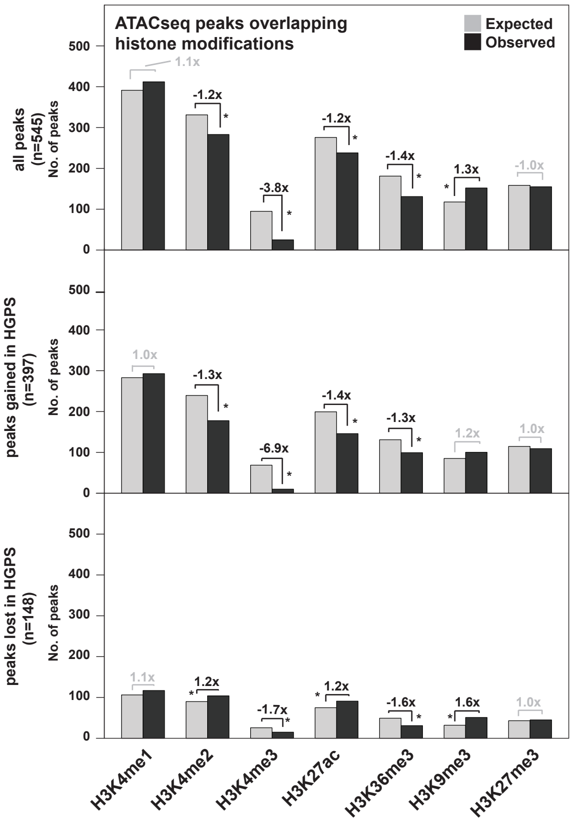

Fig. S5. Differentially accessible regions overlapping with histone modifications. Overlap of ATAC-seq peaks with the indicated histone modifications (*P<0.05, Fisher's Exact test). The expected number of peaks was calculated based on the fraction of all (including non-significant (q<0.05, Benjamini-Hochberg)) peaks overlapping with a certain histone modification normalized to the number of peaks significantly (q<0.05, Benjamini-Hochberg) gaining or losing accessibility, or both.

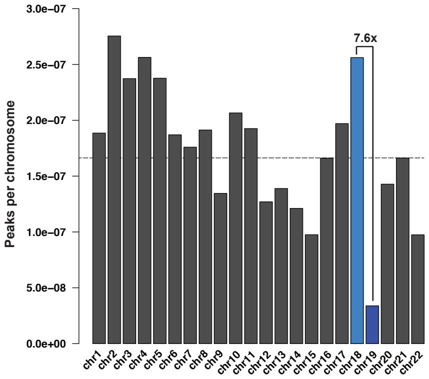

Fig. S6. Distribution of differentially accessible regions across chromosomes. Distribution of ATAC-seq peaks across chromosomes (normalized to chromosome length) with mean number indicated as a dashed line (mean=1.66e-07 peaks per chromosome). Chromosome 18 exhibited 7.6 times more differentially accessible peaks than chromosome 19.

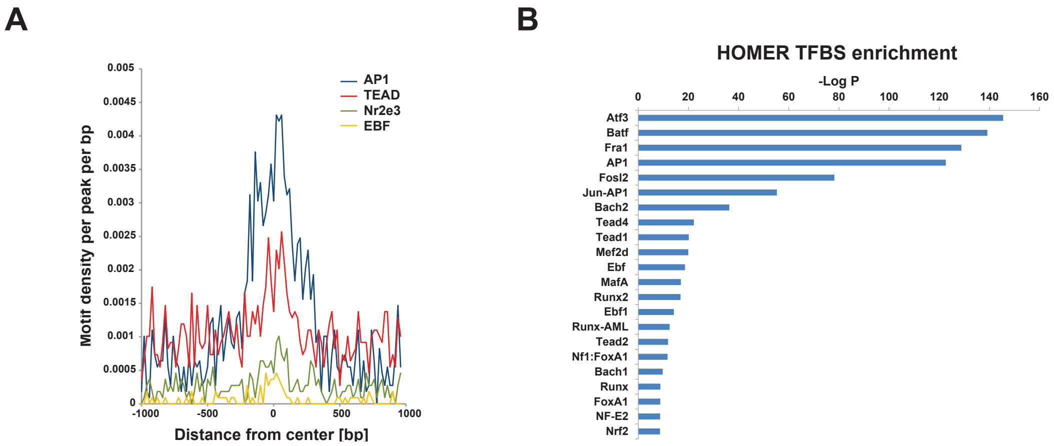

Fig. S7. AP1 family members are enriched among HGPS-specific differentially accessible regions. (A) Motif density plot shows an enrichment of AP1 family member transcription factor binding sites (TFBS) in the differentially accessible regions. Motif densities were calculated using the HOMER motif density tool for the top *de novo* motifs. **(B)** NRF2 binding motifs are among the TFBS enriched in the differentially accessible regions (NRF2: q=1.80e-3, Benjamini-Hochberg).

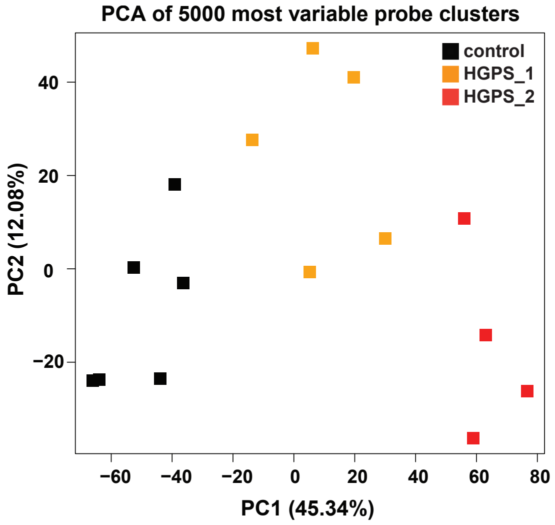

Fig. S8. DNA methylation profiling in HGPS reveals two patient subgroups. Principal component analysis (PCA) of 9 HGPS and 6 control samples using the 5,000 most variable probe clusters. The variances explained by PC1 and PC2 are given in brackets. The two HGPS subgroups identified through this analysis are indicated as HGPS_1 and HGPS_2.

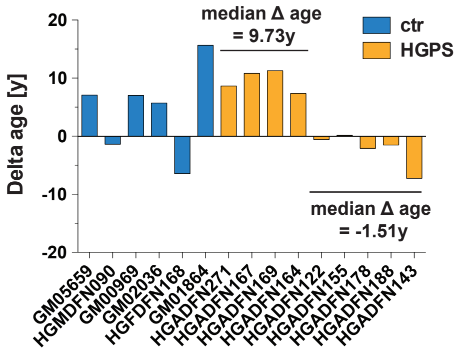

Fig. S9. Skin & Blood Clock classifies HGPS subgroups as accelerated and non-accelerated. The difference (= Δ age) between DNA methylation age (as calculated by the Skin&Blood Clock (25)) and chronological age is depicted for all samples.

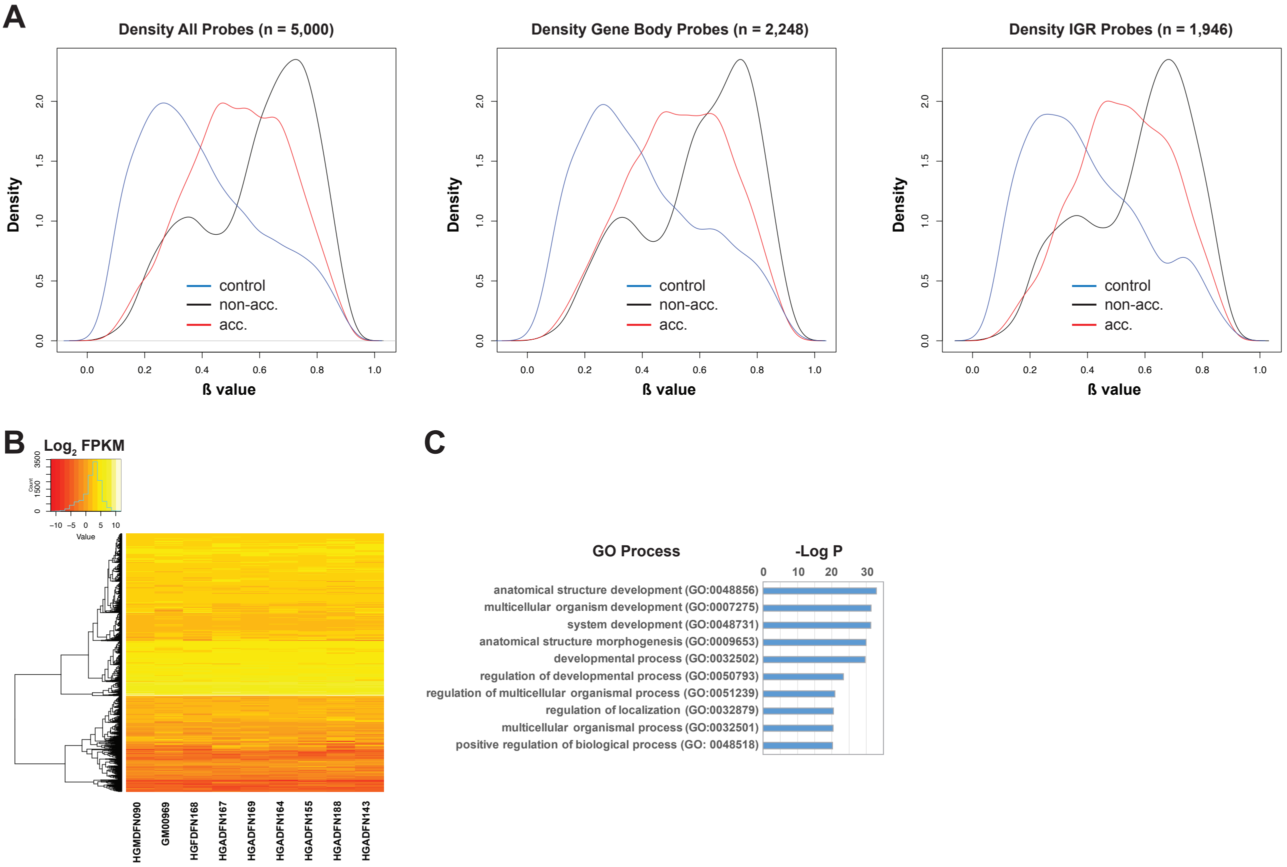

Fig. S10. The age-accelerated HGPS subgroup is characterized by partial DNA methylation (A) ß value density distribution across all (n=5,000), gene body-associated (n=2,248) or intergenic region (IGR)-associated (n=1,946) most variable probe clusters for control, accelerated and non-accelerated groups. Non-acc. = non-accelerated, acc. = accelerated. **(B)** Expression of genes (n=1,336) containing one or more of the 2,248 gene body-associated probe clusters in the sample set. **(C)** Gene Ontology (GO) processes enriched among the genes from (B).

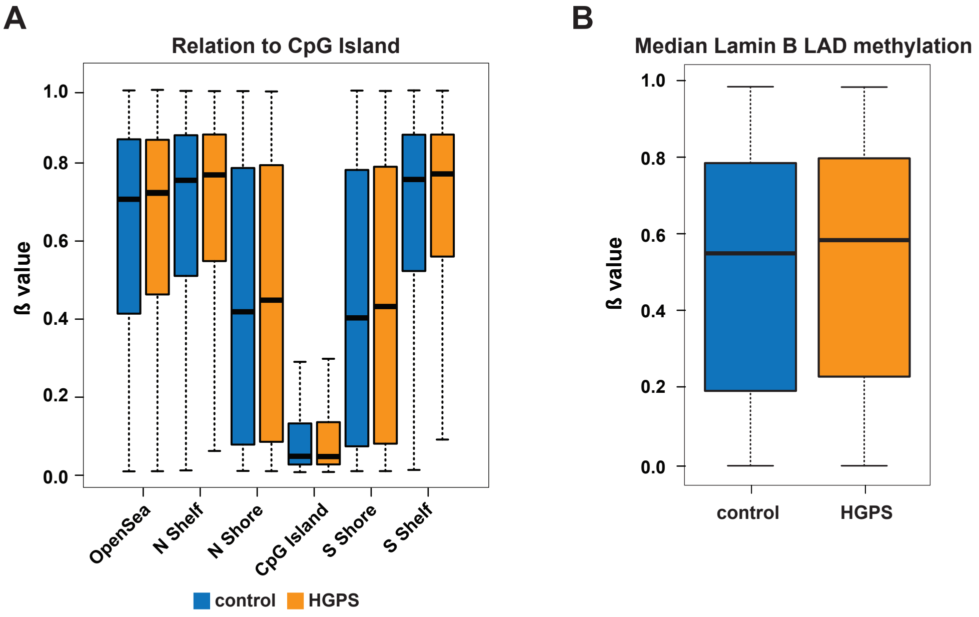

Fig. S11. DNA methylation in CpG islands and regions associated with Lamin B. (A) Median methylation (ß value) levels of OpenSea (P=1.49e−93), Shelf (N: P=3.17e−09, S: P=8.77e−08), Shore (N: P=2.5e−08, S: P=1.17e−07) and CpG Island (P=0.82)-associated probes in control (blue) and HGPS (orange) samples (all: F test, Benjamini-Hochberg). **(B)** Median methylation (ß value) levels of Lamin B LAD-associated probes in control (blue) and HGPS (orange) samples (P=2.20e-16, Welch Two Sample t-test).

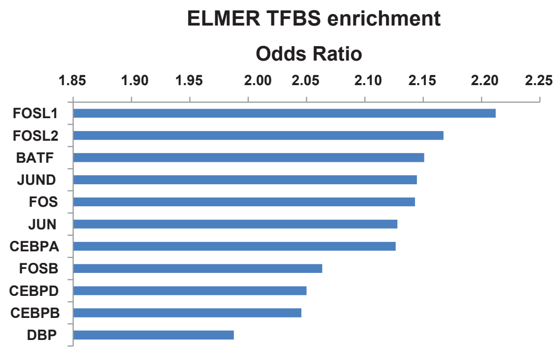

Fig. S12. AP1 family members are enriched in differentially methylated regions. ELMER transcription factor binding site (TFBS) enrichment analysis reveals that members of the AP1 family are enriched in the differentially methylated (P<0.05, F-test) regions (95% Confidence Interval).

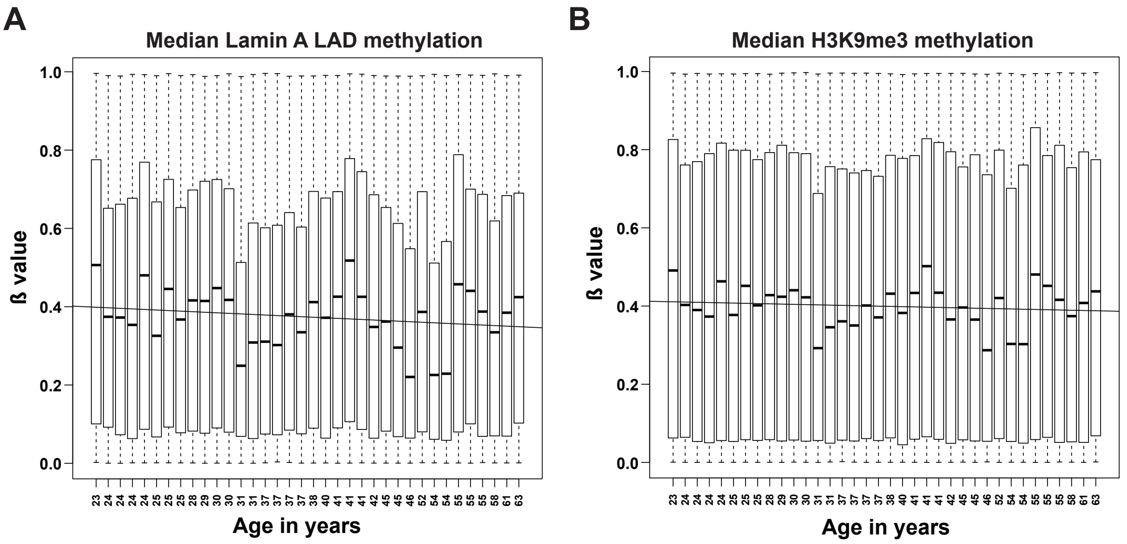

Fig. S13. LAD methylation in normal fibroblasts of different ages. (A) Median methylation (ß value) levels of Lamin A LAD-associated probes in normal fibroblasts from donors aged 23-63 years (GSE52025). Linear regression: y=-0.0014x+0.40 **(B)** As in (A), but for probes overlapping with regions marked by H3K9me3. Linear regression: y=-0.0007x+0.41.

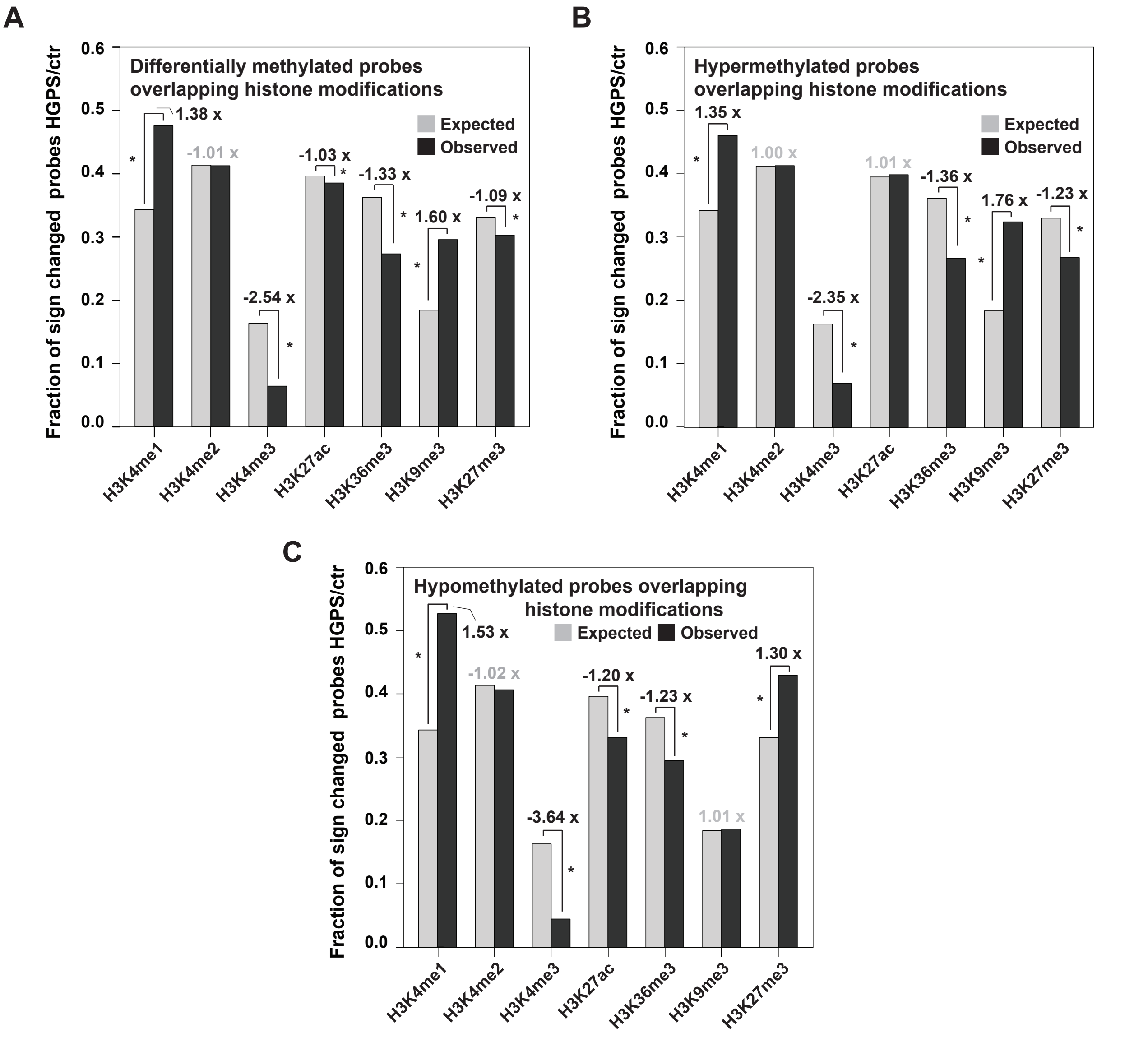

Fig. S14. Differential methylation in regions overlapping histone modifications in dermal fibroblasts. (A) Enrichment of probes overlapping with a given histone modification among the probes differentially ($P<0.05$, F test) methylated between HGPS and control samples. Fold changes as indicated. H3K4me1: P<2.20e-16, H3K4me2: P=0.77, H3K4me3: P<2.20e-16, H3K27ac: P<1.33e-03, H3K36me3: P<2.20e-16, H3K9me3: P<2.20e-16, H3K27me3: P<2.20e-16; Chi-squared test with Yates' continuity correction. Expected numbers were calculated based on the distribution of all 850,000 probes. **(B)** As in (A), but with probes hypermethylated in HGPS. H3K4me1: P<2.20e-16, H3K4me2: P=0.87, H3K4me3: P<2.20e-16, H3K27ac: P=0.36, H3K36me3: P<2.20e-16, H3K9me3: P<2.20e-16, H3K27me3: P<2.20e-16; Chi-squared test with Yates' continuity correction. **(C)** As in (A), but with probes hypomethylated in HGPS. H3K4me1: P<2.20e-16, H3K4me2: P=0.37, H3K4me3: P<2.20e-16, H3K27ac: P<2.20e-16, H3K36me3: P<2.20e-16, H3K9me3: P=0.69, H3K27me3: P<2.20e-16; Chi-squared test with Yates' continuity correction.

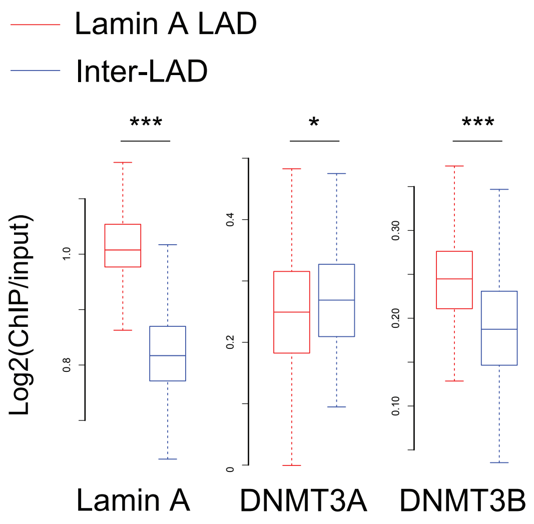

Fig. S15. DNMT3B exhibits enriched binding in Lamin A LADs. Differential enrichment of Lamin A (P<2.20e-16), DNMT3A (P=5.83e-04) and DNMT3B (P<2.20e-16) in Lamin A LADs or inter-LAD regions (Wilcoxon test for all). ChIP-seq data were obtained from a recent publication [41].

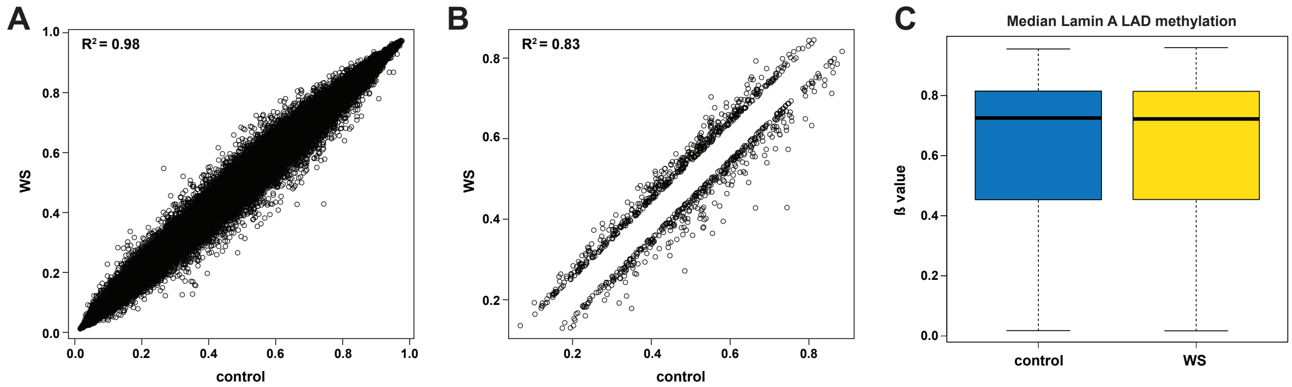

Fig. S16. Absence of Lamina-associated domain (LAD) – specific DNA hypermethylation in Werner Syndrome (WS). (A) Scatter plot comparing the methylomes of classical WS (n=18) and control (n=24) blood (GSE131752) for 812,171 probes. The coefficient of determination is given. **(B)** As in (A), but for the 1,000 probes with the highest absolute ß value differences. **(C)** Median methylation (ß value) levels of Lamin A LAD-associated probes in control (blue) and WS (yellow) samples (P=0.6534, Welch Two Sample t-test).

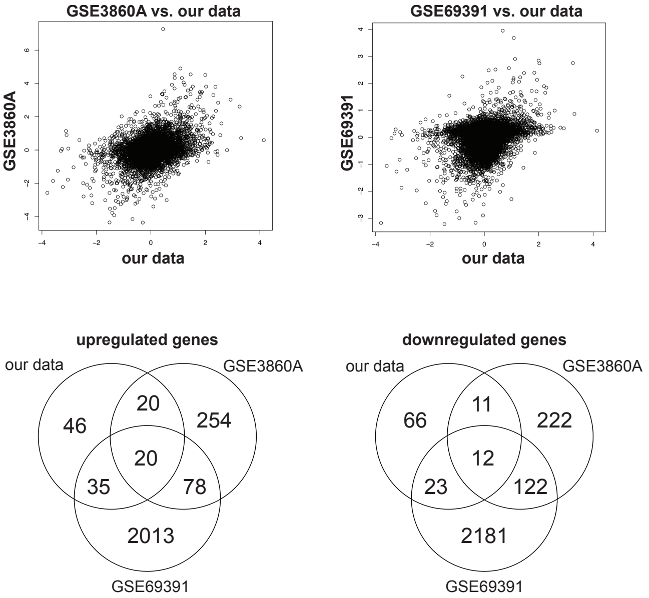

Fig. S17. Comparison of RNAseq data with previous HGPS expression studies. Log Fold Changes (FC) of genes in GSE3860A vs. our data (Pearson correlation r=0.37, P<2.20e-16), and in GSE69391 vs. our data (Pearson correlation r=0.23, P<2.20e-16), respectively, are given in upper panels. Lower panels show the overlap of genes up- or downregulated, respectively, between our and the aforementioned studies.

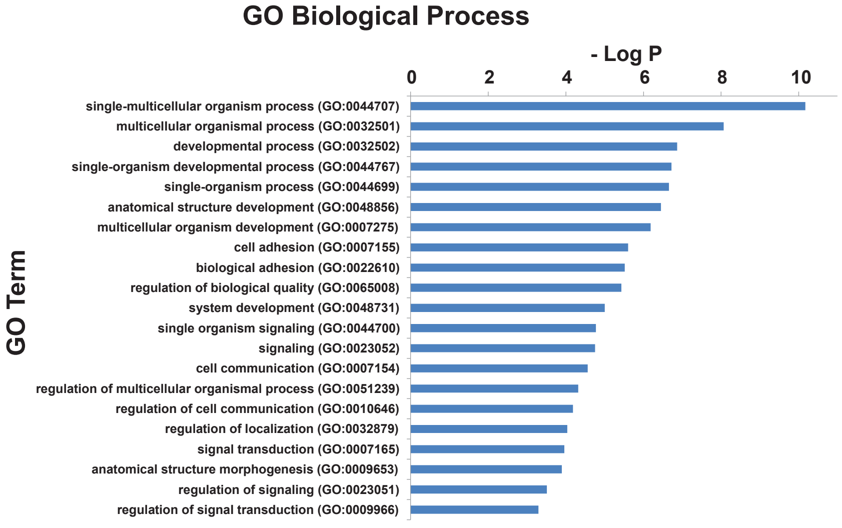

Fig. S18. Organismal and developmental processes are enriched in the differentially expressed genes in HGPS. Gene Ontology (GO) processes enriched among the differentially ($q<0.05$, Benjamini-Hochberg) expressed genes in HGPS.

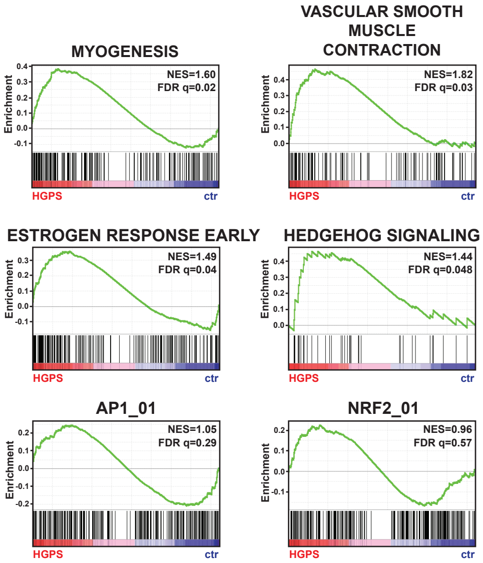

Fig. S19. Gene Set Enrichment Analyses (GSEAs) of additional processes enriched in the HGPS-specific transcriptome. Additional hallmark and Kyoto Encyclopedia of Genes and Genomes (KEGG) gene sets enriched (FDR q<0.05) in HGPS fibroblasts, plus GSEA analyses of AP1 and NRF2 target genes, respectively.

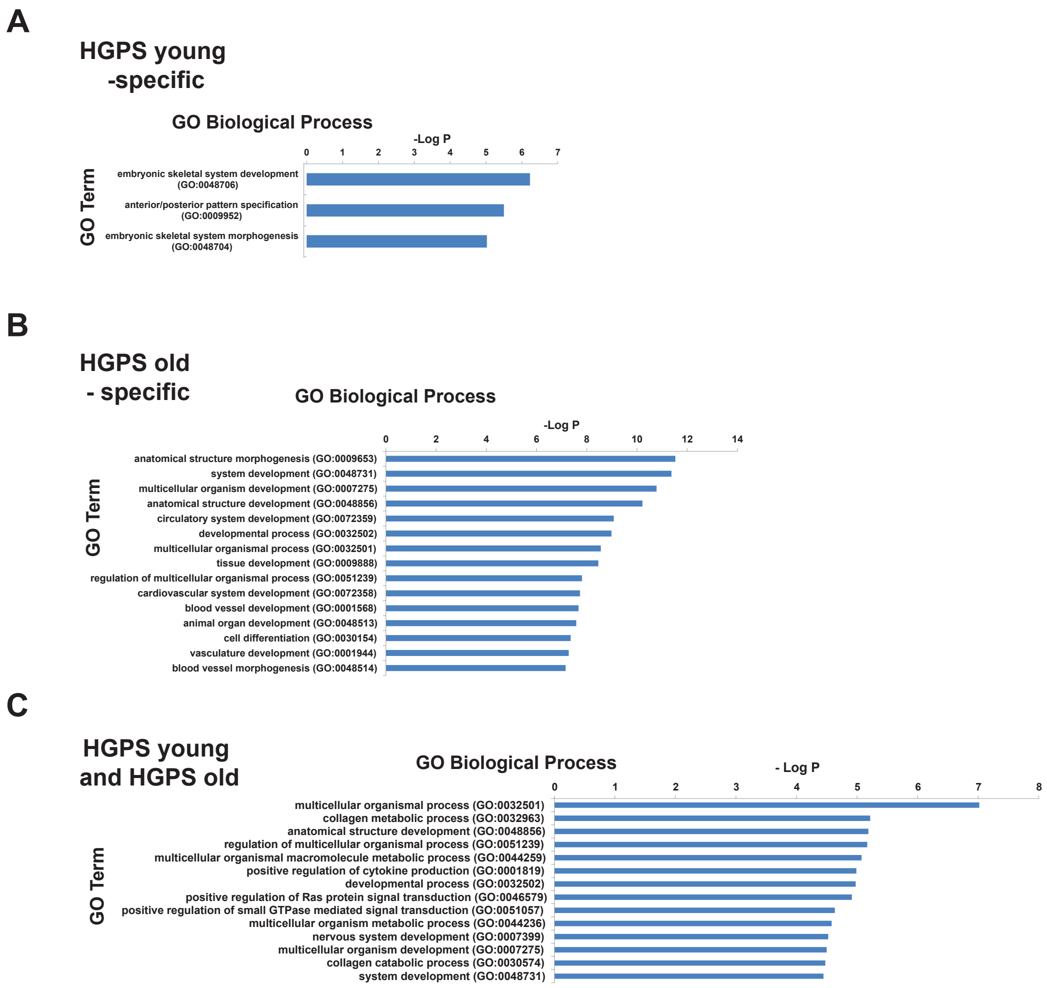

Fig. S20. Gene Ontology (GO) processes enriched in the different HGPS age groups. GO processes enriched among the differentially (q<0.05, Benjamini-Hochberg) expressed genes for **(A)** HGPS young (<8 years) vs. control, **(B)** HGPS old (>8 years) vs. control and **(C)** genes overlapping between HGPS old vs. control and HGPS young vs. control, respectively.

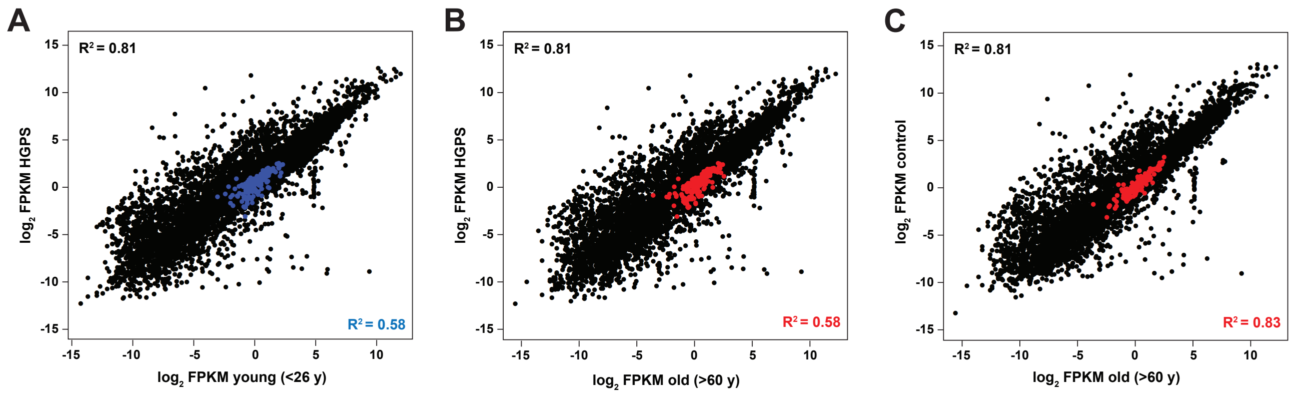

Fig. S21. HGPS fibroblast expression patterns differ from those of old normal fibroblasts. Scatter plots showing the correlation in expression of genes (n=9,630) in HGPS fibroblasts vs. fibroblasts from young (<26 years) donors (n=36) in **(A)**, in HGPS fibroblasts vs. fibroblasts from old (>60 years) donors (n=47) in **(B)**, and in control fibroblasts vs. fibroblasts from old (>60 years) donors (n=47) in **(C)**. Genes identified to be differentially expressed in HGPS (n=343) and their coefficients of determination are given in colors. Normal fibroblast expression data were obtained from GSE113957. FPKM = Fragments Per Kilobase of transcript per Million mapped reads, y = years.

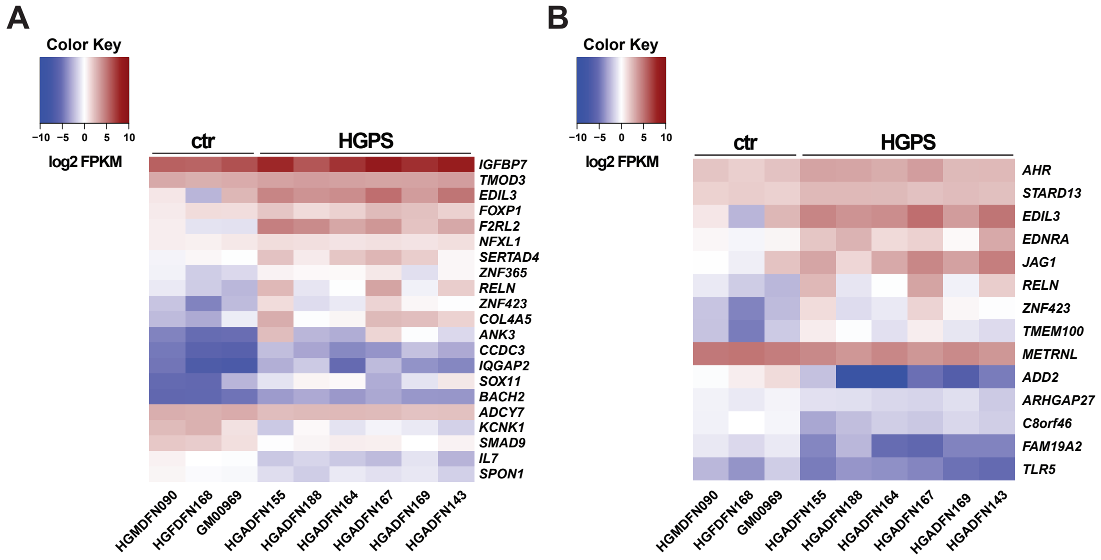

Fig. S22. Epigenetic deregulation of lamina-associated domains (LADs) contributes to aberrant gene expression in HGPS. (A) Differentially (q<0.05, Benjamini-Hochberg) expressed genes that show concomitant HGPS-specific chromatin accessibility changes in their promoter or gene body. Lowly expressed genes are shown in blue, highly expressed ones in red. FPKM = Fragments Per Kilobase of transcript per Million mapped reads. **(B)** Differentially (q<0.05, Benjamini-Hochberg) expressed genes that show concomitant HGPS-specific DNA methylation changes in their promoter or gene body. Lowly expressed genes are shown in blue, highly expressed ones in red. FPKM as in (A).

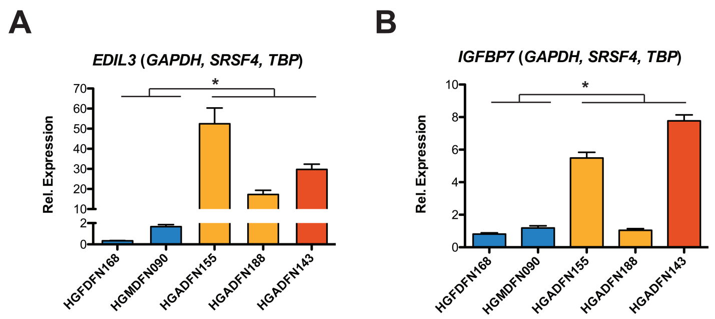

Fig. S23. Overexpression of *EDIL3* and *IGFBP7* in HGPS fibroblasts as verified by RT-qPCR. (A) and **(B)** *EDIL3* and *IGFBP7* expression levels relative to those of *GAPDH*, *SRSF4* and *TFB* in control (blue), HGPS young (<8 years, orange) and HGPS old (>8 years, red) cells as measured in RT-qPCR (**EDIL3*: P=2.00e-3, *IGFBP7*: P=4.00e-4, unpaired t-test).

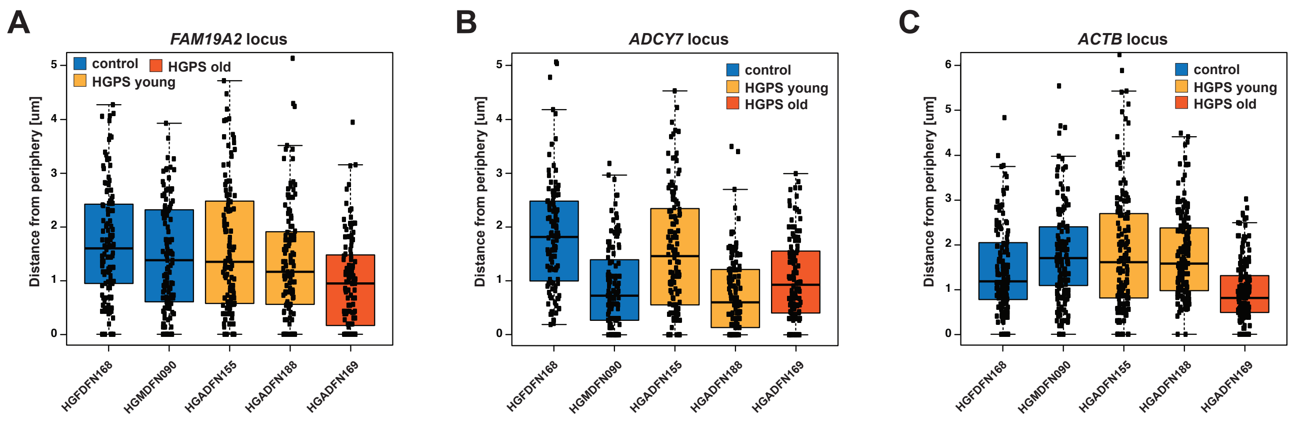

Fig. S24. Quantification of distance from nuclear periphery to gene locus for additional genes. (A), **(B)** and **(C)** Quantification of the distance from the FISH signal to the nuclear periphery for *FAM19A2*, *ADCY7* and *ACTB* loci in two control and three HGPS cell lines for 60 cells per sample. *FAM19A2*: P=1.13e-03; *ADCY7*: P=1.62e-03; *ACTB*: P=0.20, Welch Two Sample t-test for all.

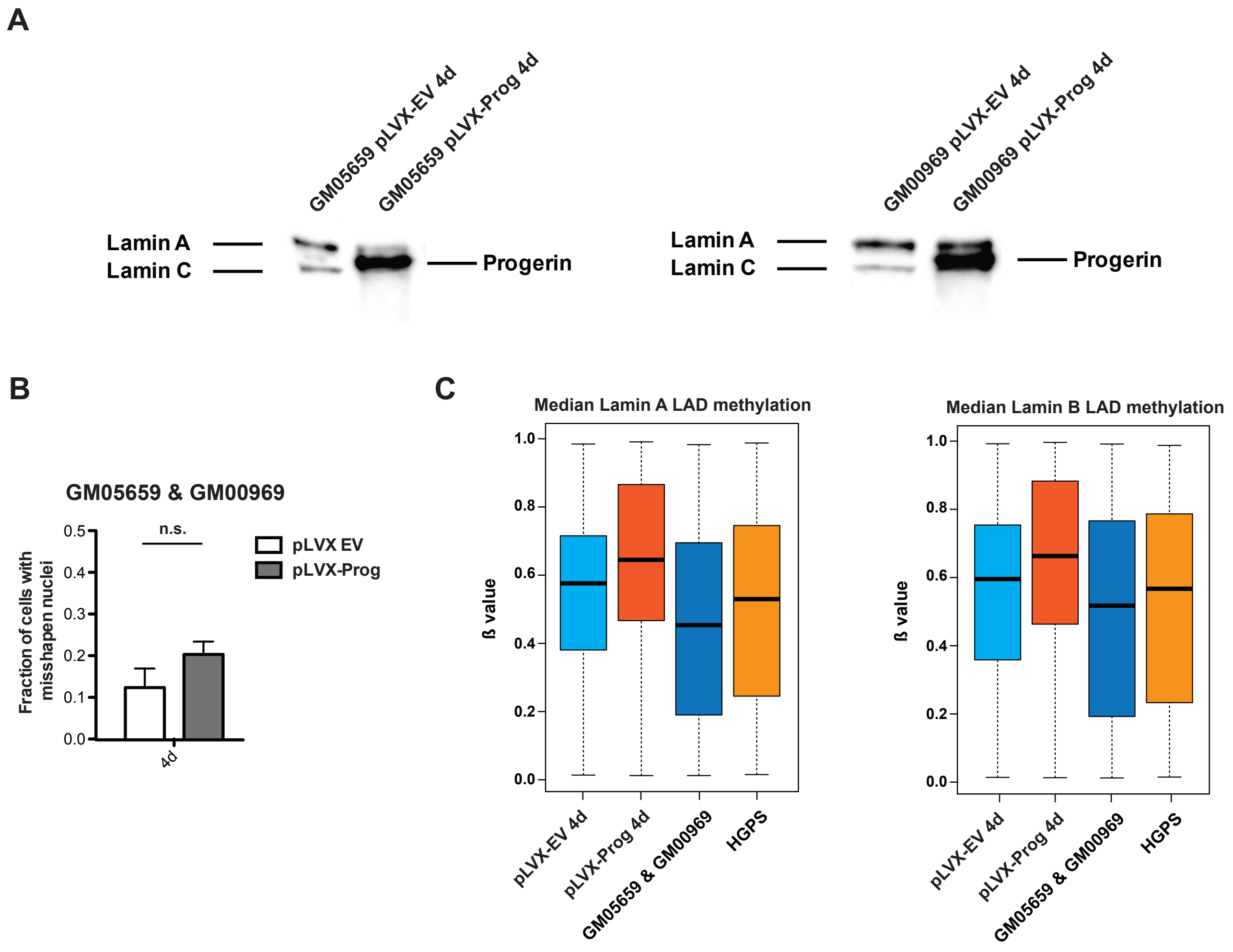

Fig. S25. Ectopic expression of Progerin in control fibroblasts. (A) Western blot with α-Lamin A/C antibody (sc7292, Santa Cruz) showing expression of Progerin in two fibroblast cell lines transduced with pLVX-Prog 4 days after transduction. **(B)** Fractions of misshapen nuclei in pLVX-EV- or pLVX-Prog-transduced fibroblasts 4 days after transduction, as measured by α-Lamin A/C immunofluorescence (triplet measurements for each cell line). An average from both transduced cell lines is shown. **(C)** Median methylation (ß value) levels of Lamin A and Lamin B LAD-associated probes in pLVX-EV- (light blue) or pLVX-Prog-transduced (red) fibroblasts (Lamin A LADs: $P<2.20e-16$, Lamin B LADs: $P<2.20e-16$, both: Welch Two Sample t-test), as well as in control (dark blue) or HGPS (orange) fibroblasts. For pLVX-EV 4d and pLVX-Prog 4d, the averages of two transduced cell lines are shown.

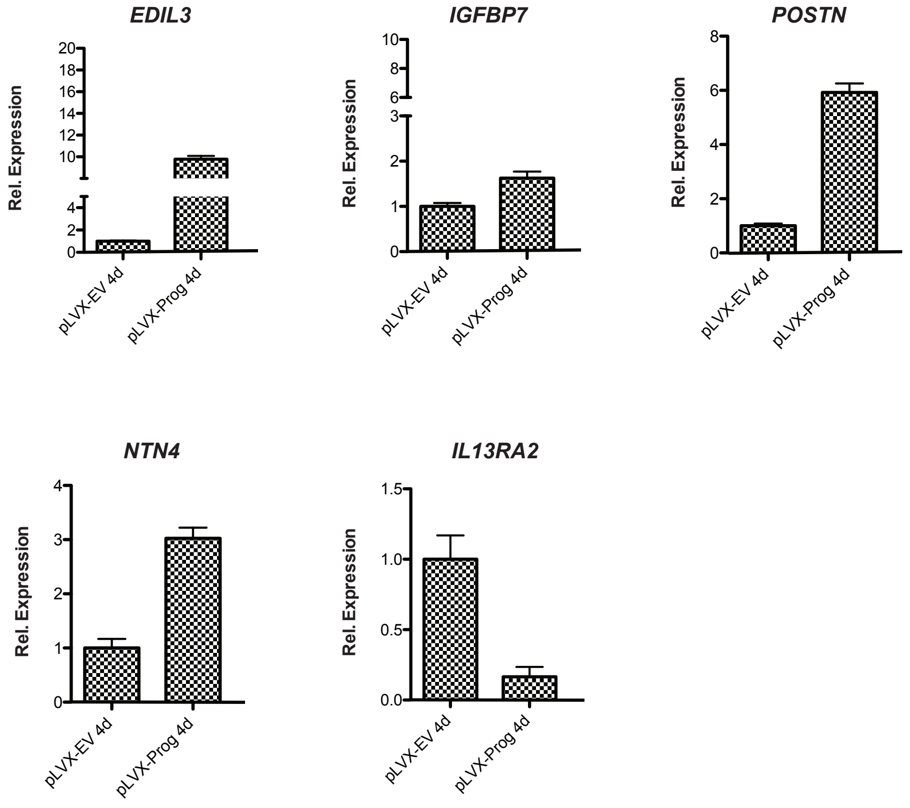

Fig. S26. Extopic expression of Progerin causes gene expression changes similar to those of HGPS cells. *EDIL3*, *IGFBP7*, *POSTN*, *NTN4* and *IL13RA2* expression levels relative to those of *GAPDH*, *SRSF4* and *TFB* in pLVX-EV- or pLVX-Prog-transduced fibroblasts, 4 days after transduction, as measured by RT-qPCR. Averages of both transduced cell lines are shown.
