# Supplementary material for: Epigenetic deregulation of lamina-associated domains in Hutchinson-Gilford progeria syndrome
Source: Genome Med. 2020 May 25;12:46. doi: 10.1186/s13073-020-00749-y (PMC7249329; doi:10.1186/s13073-020-00749-y)

Original Western Blot with Lamin A/C antibody (sc7292, Santa Cruz) used for Fig. S2: Progerin expression in the fibroblast model system. The lower row was used for illustrating Progerin expression in HGPS cells in Fig.S2.

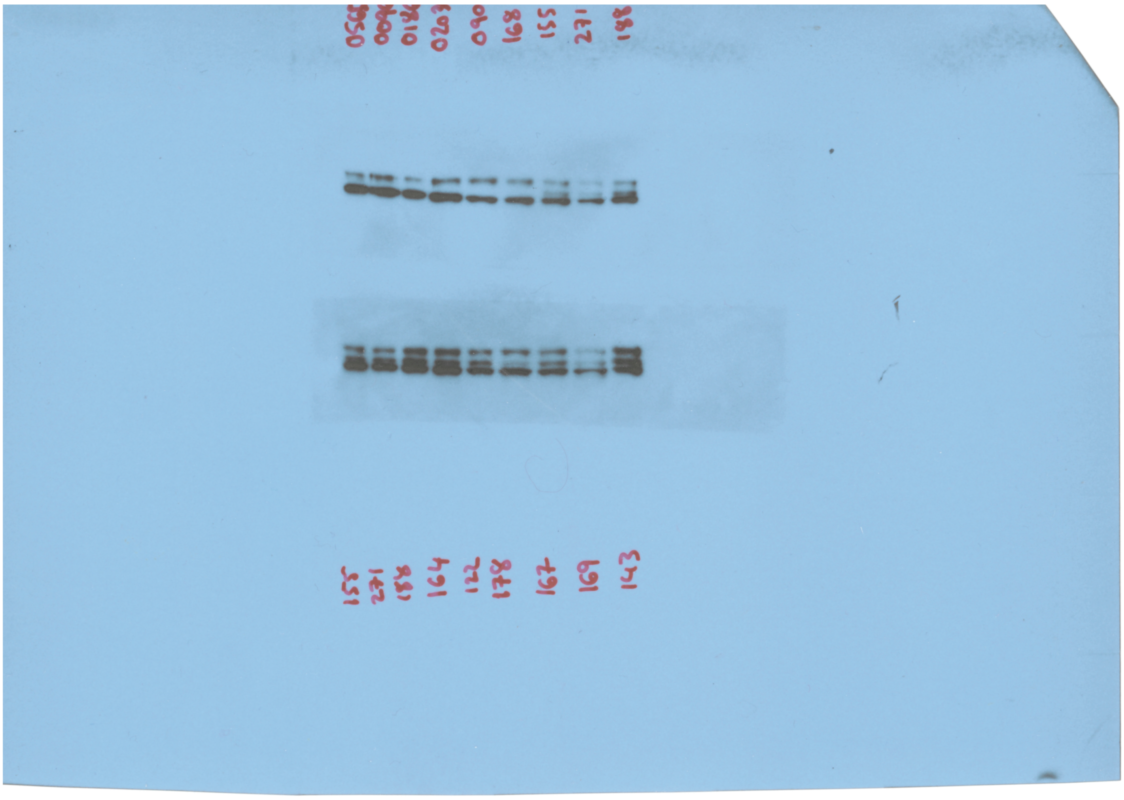

Original Western Blot with Lamin A/C antibody (sc7292, Santa Cruz) used for Fig. S2: Progerin expression in the fibroblast model system. The upper row was used for illustrating Progerin expression in control cells in Fig.S2.

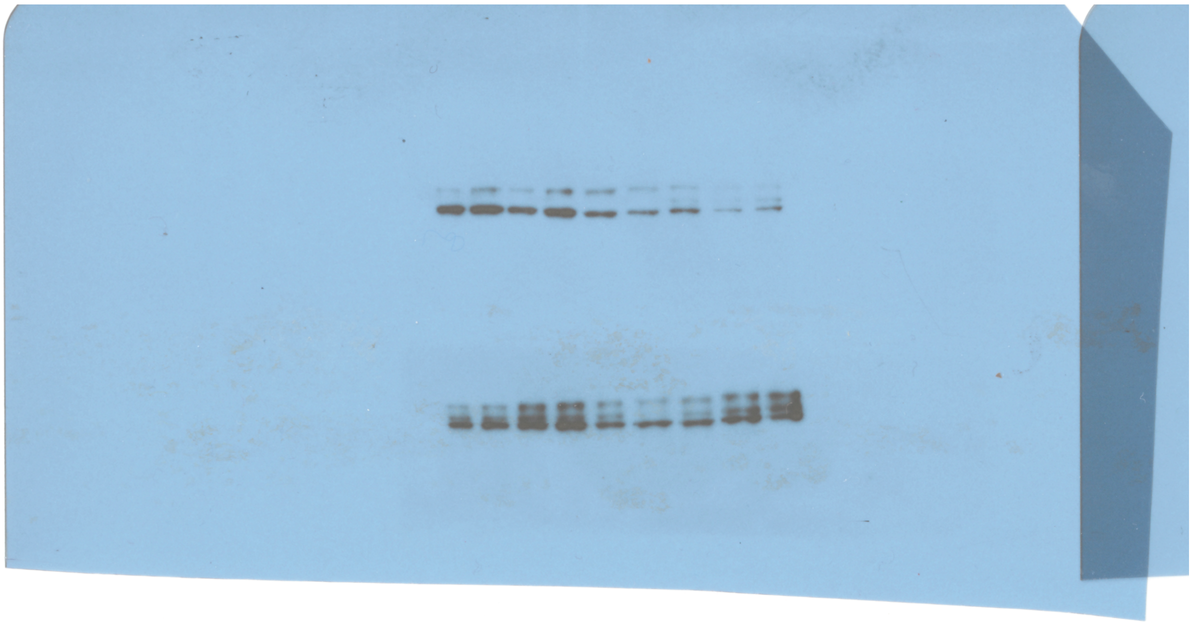

Original Western Blot with Lamin A/C antibody (sc7292, Santa Cruz) including molecular weight markers, for reference.

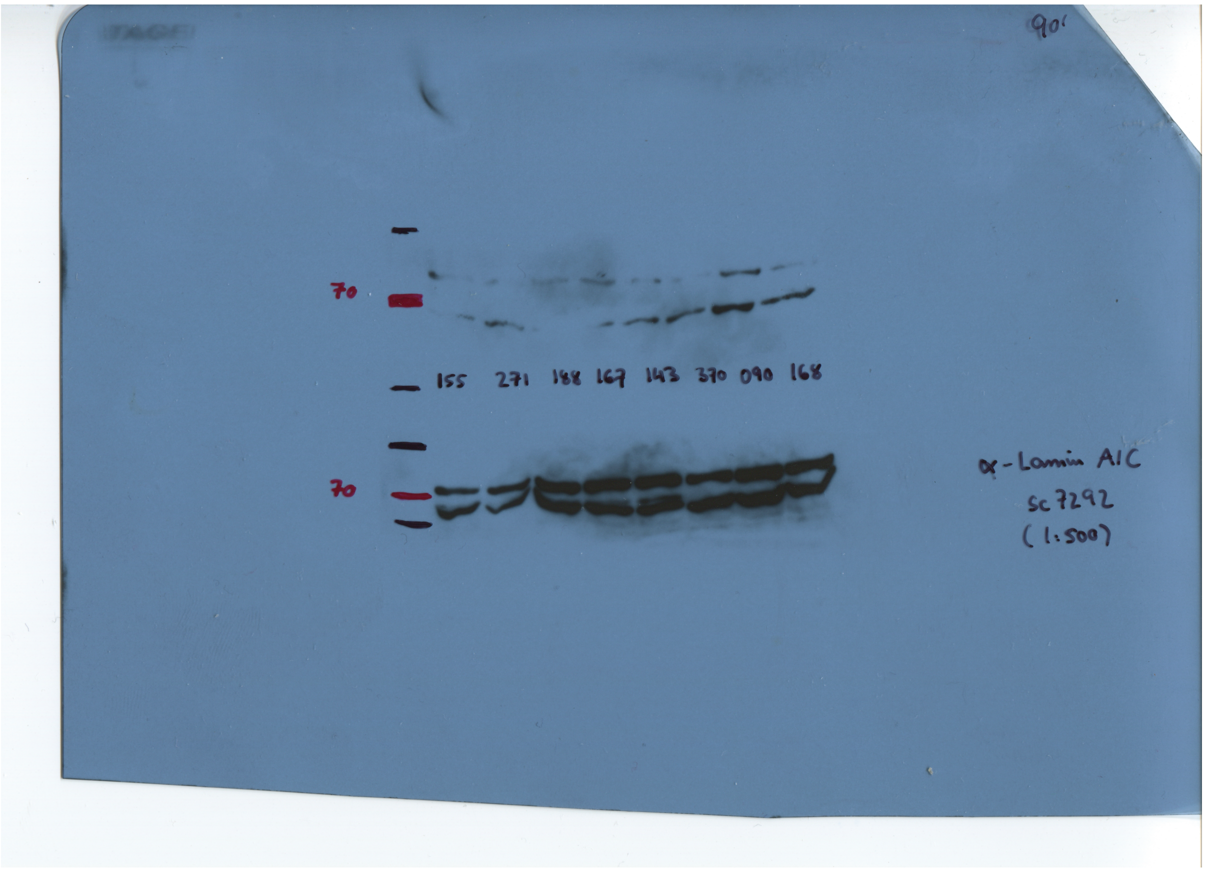

Original Western Blot with Lamin A/C antibody (sc7292, Santa Cruz) used for Fig. S25: Ectopic expression of Progerin in control fibroblasts. The upper panel shows the scan that was used for illustrating Progerin expression in control cells in Fig. S25, while the middle panel shows a longer exposure of the same membrane. The lower panel shows a photo of the same membrane including the molecular weight markers (PageRuler™ Prestained Protein Ladder, Thermo Fisher Scientific) as a reference. Marker bands from top to bottom: 130 kDa, 100 kDa, 70 kDa, 55 kDa.

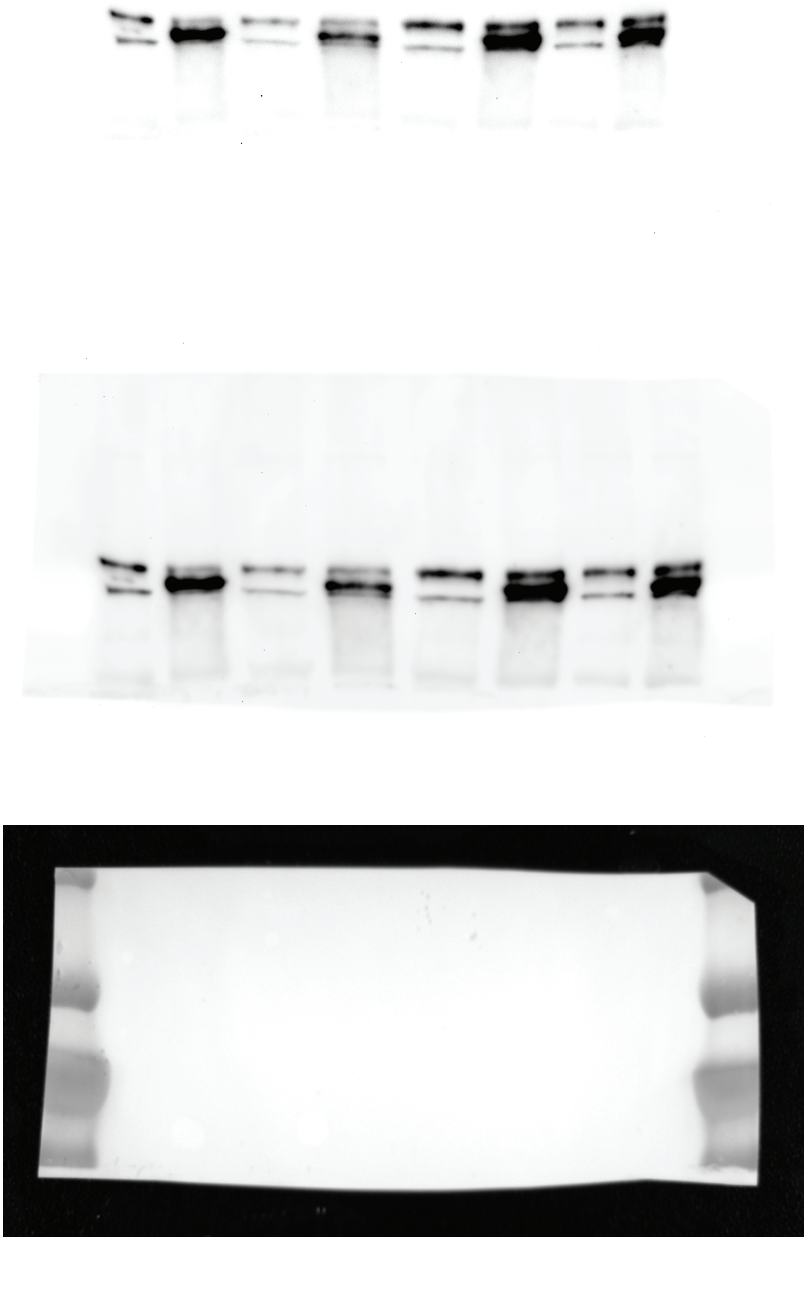

Supplement: Supplementary file 2 — Additional file 2. Original uncropped Western blots. [file 13073_2020_749_MOESM2_ESM.pdf]
